# The use of genome wide association methods to investigate pathogenicity, population structure and serovar in *Haemophilus parasuis*

**DOI:** 10.1186/1471-2164-15-1179

**Published:** 2014-12-24

**Authors:** Kate J Howell, Lucy A Weinert, Roy R Chaudhuri, Shi-Lu Luan, Sarah E Peters, Jukka Corander, David Harris, Øystein Angen, Virginia Aragon, Albert Bensaid, Susanna M Williamson, Julian Parkhill, Paul R Langford, Andrew N Rycroft, Brendan W Wren, Matthew T G Holden, Alexander W Tucker, Duncan J Maskell, on behalf of the BRADP1T Consortium

**Affiliations:** Department of Veterinary Medicine, University of Cambridge, Madingley Road, Cambridge, CB3 0ES UK; Department of Molecular Biology and Biotechnology, University of Sheffield, Firth Court, Western Bank, Sheffield, S10 2TN UK; Department of Mathematics and Statistics, University of Helsinki, Helsinki, 00100 Finland; The Wellcome Trust Sanger Institute, Wellcome Trust Genome Campus, Hinxton, Cambridge CB10 1SA UK; Norwegian Veterinary Institute, N-0106 Oslo, Norway; Centre de Recerca en Sanitat Animal (CReSA), UAB-IRTA, Campus de la Universitat Autònoma de Barcelona, 08193, Bellaterra, and, Institut de Recerca i Tecnologia Agroalimentàries (IRTA), Barcelona, Spain; Animal Health and Veterinary Laboratories Agency (AHVLA), Rougham Hill, Bury St Edmunds, Suffolk, IP33 2RX UK; Department of Medicine, Section of Paediatrics, Imperial College London, St. Mary’s Campus, London, W2 1PG UK; The Royal Veterinary College, Hawkshead Campus, Hatfield, AL9 7TA Hertfordshire, UK; Faculty of Infectious & Tropical Diseases, London School of Hygiene & Tropical Medicine, Keppel Street, London, WC1E 7HT UK; School of Medicine, University of St. Andrews, St Andrews, KY16 9TF UK

**Keywords:** Genome wide association study, Pan-genome, Recombination, DAPC, Virulence factors, Population structure, *Haemophilus parasuis*

## Abstract

**Background:**

*Haemophilus parasuis* is the etiologic agent of Glässer’s disease in pigs and causes devastating losses to the farming industry. Whilst some hyper-virulent isolates have been described, the relationship between genetics and disease outcome has been only partially established. In particular, there is weak correlation between serovar and disease phenotype. We sequenced the genomes of 212 isolates of *H. parasuis* and have used this to describe the pan-genome and to correlate this with clinical and carrier status, as well as with serotype*.*

**Results:**

Recombination and population structure analyses identified five groups with very high rates of recombination, separated into two clades of *H. parasuis* with no signs of recombination between them. We used genome-wide association methods including discriminant analysis of principal components (DAPC) and generalised linear modelling (glm) to look for genetic determinants of this population partition, serovar and pathogenicity. We were able to identify genes from the accessory genome that were significantly associated with phenotypes such as potential serovar specific genes including capsule genes, and 48 putative virulence factors that were significantly different between the clinical and non-clinical isolates. We also show that the presence of many previously suggested virulence factors is not an appropriate marker of virulence.

**Conclusions:**

These genes will inform the generation of new molecular diagnostics and vaccines, and refinement of existing typing schemes and show the importance of the accessory genome of a diverse species when investigating the relationship between genotypes and phenotypes.

**Electronic supplementary material:**

The online version of this article (doi:10.1186/1471-2164-15-1179) contains supplementary material, which is available to authorized users.

## Background

*Haemophilus parasuis* is an important pathogen of pigs [1, 2] but is also a frequent commensal of their upper respiratory tracts. It causes polyserositis, meningitis and septicaemia (known as Glässer’s disease) as well as pneumonia and pleurisy [3]. Historically*, H. parasuis* caused disease in recently weaned piglets, but with the intensification of production it now affects a wide age-range [4, 5]. *H. parasuis* is the leading cause of mortality, alongside the PRRS virus, in nursery herds in the USA, and is the third-most important bacterial pathogen affecting finisher herds [6]. *H. parasuis* also contributes to multi-factorial porcine respiratory disease complex, the leading cause of mortality in grower-finisher pigs in the USA [7]. *H. parasuis* is endemic in all pig farming countries and has a major economic impact on the global pig industry, with costs including those for production and stock losses, carcass disposal, and vaccination, as well as requiring antibiotic therapy [8–10].

The study of *H. parasuis* to date has classified the bacterium, originally using a gel immuno-diffusion assay and more commonly now an indirect haemagglutination assay, into 15 different serovars with non-typeable isolates also frequently found [11, 12]. The designation of serovar in *H. parasuis* is thought to be predominantly due to the presence of a particular polysaccharide capsule which is specific to each serovar (based on the reference strains) [13–16]. Some serovars, especially 4, 5 and 13, cause disease more commonly than others [17–20]. However, no absolute relationship between virulence and serovar has been found [21, 22]. The onset of disease has been linked to many factors, including physiological stress and immune status, but the current study focuses on understanding the bacterial genetic factors that might correlate with the establishment of disease.

Several experiments have tried to associate the bacterium’s genotype with phenotype, but with limited success [23–29]. Experimental evidence to support definitive virulence factors in *H. parasuis* is sparse and most studies have been limited to small numbers of isolates or serovars. It is likely that the relationship between genotype and virulence phenotype is complex [2, 30–39]. The first *H. parasuis* genome to be sequenced was in 2010 [40], with several further sequences released since then [41, 42]. These have been used to design several experiments, including searches for novel immunogenic proteins [43] and virulence factors [38, 44], but these few strains may not be reflective of the wider diversity of isolates that exist in pig populations. Previous studies show that the pan-genome of a bacterial species can be vast [45–49]. Recombination and lateral transfer are involved in creating the pan-genome, which can be resolved into the core and accessory genomes. The boundaries of the core genome can be extrapolated from a small number of genomes, but it is preferable to analyse a large number of isolates for a more comprehensive definition [50–52]. An extensive amount of variation can often be found within the accessory genome of a species and it is important to investigate this source of variation to understand the adaptive potential of a given species [48, 53]. Reductions in cost have allowed the whole genome sequencing of many strains of the same species, and this can provide the necessary statistical power required to find associations between genotypes and phenotypes such as virulence. This variation is now being explored in many pathogens including *Campylobacter jejuni*[54]*, Escherichia coli*[48, 55, 56], *Haemophilus influenzae*[57]*, Staphylococcus aureus*[58] and *Streptococcus pneumoniae*[59]. Such studies seek associations between phenotypes and genotypes that would inform surveillance efforts, improve diagnostics, and aid understanding of the pathogen, potentially yielding new treatments of the associated diseases.

In order to disentangle the genetic and environmental bases of Glässer’s disease, we undertook a pan-genome analysis of 212 isolates of *H. parasuis* with high quality clinical metadata, covering all serovars and isolates from disease- and non-disease-causing backgrounds. We have investigated the key features of the pan-genome and assessed the relationship between the genetics of this bacterium and collated metadata, using statistical analyses of single nucleotide polymorphisms (SNPs) in the core genome, and the presence of genes in the accessory genome. We compared our analyses to the distribution of previously published virulence factors of *H. parasuis* within our collection of isolates. We found that the composition of the accessory genome was a significant factor in determining whether isolates were likely to cause disease. Our findings emphasise the importance of describing the pan-genome of a bacterial species in order to understand pathogenesis, and to underpin a new generation of vaccine and diagnostic candidates for endemic bacterial pathogens.

## Results and discussion

### Epidemiology of the isolate collection

The 212 isolates in the collection originate from the UK (n = 121 from 70 geographically dispersed farms), Argentina (n = 1), Belgium (n = 1), China (n = 2), Denmark (n = 29), Germany (n = 6), Greece (n = 1), Italy (n = 2), Japan (n = 7), Spain (n = 21), Sweden (n = 4), Switzerland (n = 2) and the USA (n = 6), with nine isolates of unknown origin. Our analysis included the reference strains of the 15 serovars and the published genomes of the US strain 29755, and the Chinese strains SH0165 and ZJ0906 [40, 41, 60]. The dates of collection ranged between 1991–2008 for European isolates and 1955–1992 for the reference strains [61]. For the UK isolates, we collected clinical metadata including veterinary diagnostic information from necropsy, the tissue of isolation and details of any other signs of disease in the pig. Clinical metadata for the other strains included tissue of origin, clinical signs and, for a subset of strains, experimental reproduction of disease. We used this information to categorise the isolates into clinical and non-clinical categories. A non-clinical isolate was an isolate identified in the upper respiratory tract in the absence of any signs of respiratory or systemic disease typical of infection with *H. parasuis.* A clinical isolate was an isolate identified from an animal displaying signs of *H. parasuis*-related disease and isolated from a respiratory or a systemic site. We divided the clinical isolates further into respiratory and systemic, to reflect disease severity. Respiratory isolates were obtained from the lung without signs of systemic disease elsewhere in the animal. Systemic isolates were obtained from a systemic site e.g. brain, joint or blood which are sites typically affected in Glässer’s disease. Isolates that were taken from the lung of an animal that also showed signs of systemic disease were first tested in the statistical analyses as respiratory isolates and then as systemic. This was to allow for the possibility of these isolates being from a co-infection in the same animal or where the same isolate caused disease elsewhere in the same animal.

**Figure 1:**
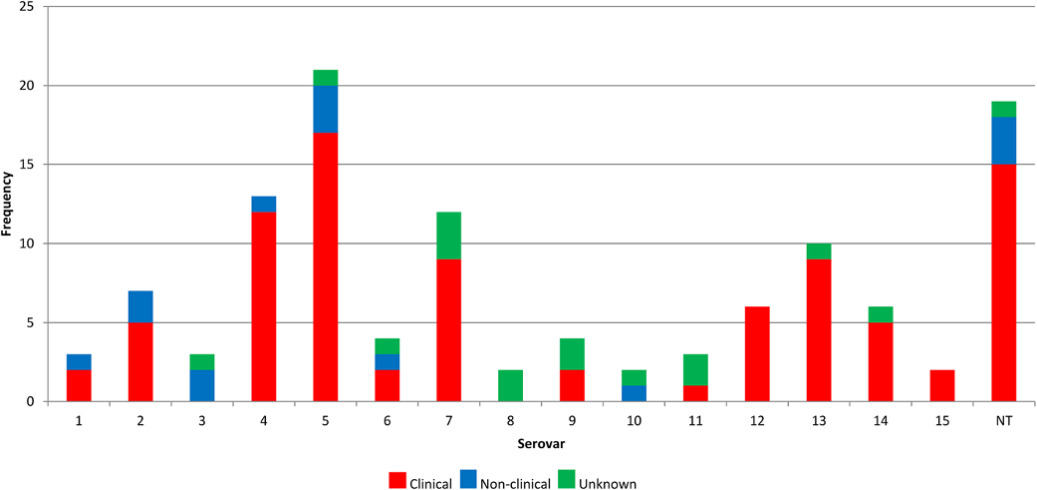
***H. parasuis*** **isolate collection displayed by serovar and disease.** Each serovar includes those found with cross-reactions. NT denotes 'non-typeable’ by serotyping either due to no reaction or due to more than three reactions to the serotyping antisera. Strains without serotyping data are excluded.

One hundred and seventeen of the isolates had been previously serotyped (including 15 that were deemed non-typeable) and these included examples of all fifteen known serovars. For details of the isolate collection including disease categories and serotyping results see Additional file 1: Table S1. Analysis of the distribution of isolates by serovar and disease association (Figure 1) showed that the most prevalent serovar in this collection was serovar 5, followed by non-typeables (NT) and serovars 4 and 7, which fits with global prevalence data [17–19]. Our data support the previous limited studies that show that certain serovars, particularly 4 and 5, were more strongly linked to disease [18, 61]. While serovar is still important for the formulation of vaccine strategy against this bacterium, its relationship with virulence has long been discussed but never proven [29, 61]. The fact that there are non-clinical isolates within the same serovars highlights the limitation and uncertainty of serotyping as a technique for predicting disease potential. We expect that there are isolates amongst the non-clinical collection that will have the potential to cause clinical disease given a different host or environment, and also that some of the clinical isolates caused disease due to other environmental factors or co-infections. The main reason for this study was to investigate the balance between the host, environment and genotypes for example serovar determining factors, predicted virulence genes and to discover new gene associations with phenotypes such as virulence. We propose that molecular epidemiological markers that relate to severity of disease would be more useful for *H. parasuis* surveillance now that rapid genome sequencing of isolates is possible. To achieve this goal we undertook pan-genome and statistical analyses with the two-fold aim of identifying genes that correlate with virulence and so could be worth investigating as markers of disease-causing potential, and with serovar to allow the design of a molecular-based serotyping technique.

### The pan-genome of *H. parasuis*

We use the term pan-genome to refer to the set of all of the genes predicted from every isolate in the collection. The pan-genome comprised homology groups encoding 7,431 genes from the OrthoMCL analysis of the 212 isolates. Of these, 1,049 genes were found within all isolates and so were designated as the core genome. This core genome is approximately 0.9 Mb of the 2.2 Mb average genome size with 65,137 SNPs identified. The accessory genome was made up of 6,382 homology groups that varied between isolates. 329 of these homology groups were classed as pseudogenes (with a minimum of 18 pseudogenes identified per isolate), and 28.9% of these homology groups were identified in less than 1% of the isolates in the collection. For details of the COG functional classification of the pan- genome see Additional file 2 and Additional file 3: Figure S1.

### Synteny of the pan-genome

The syntenic pan-genome of *H. parasuis* is the pan-genome reordered to reflect the chromosomal location of each gene based on the SH0165 strain [40]. Genes were inserted from contigs in descending size order, based on their order along each contig. As an iterative process not all genes could be assigned to a location in the syntenic pan-genome. Therefore the syntenic pan-genome of *H. parasuis* represents the 5,574 genes out of the total genes where the position could be identified in the pan-genome order, and is shown in Additional file 4: Figure S2. Several regions of variation can be seen within Additional file 4: Figure S2 with the location of many phage genes, highly variable regions and antibiotic resistance genes denoted by colouring of the heat-map. The capsule genes did not cluster in one location in the syntenic pan-genome, which may be due to recombination in the genomes, and so the synteny of the capsule locus was established separately and can be seen in Additional file 5: Figure S3. Several phages were identified within this collection including the Mu phage (regions 2, 3, 4 and 6), a siphovirus (which is predominantly found in the reference strains) as well as other phage genes throughout the pan-genome. For details of genes identified within these variable regions see Additional file 2. Briefly, within these regions we identified many phage genes, two conjugal transfer operons, adhesins, toxins, iron-sulphur binding proteins and many proteins of unknown function. Based on the similarity of the G + C content to the average for this bacterium, many of the genes in region 1 appear to be from *H. parasuis* genes that have yet to be characterised and submitted to NCBI*.* Regions 2, 3 and 4 contained some genes of relatively high G + C content (40-50%) but no hits were found when BLAST analysis was performed for these encoded proteins using the NCBI non-redundant protein database (nr). Only region 6 contained multiple proteins with hits from nr to other bacterial species including *Actinobacillus, Gallibacterium, Klebsiella and Mannhaimia.* The average G + C content of the pan-genome was 39.4% G + C content (±5.4%, which is the standard deviation)*,* ranging between 17.8-67.1% G + C content for all isolates, which is discussed further in Additional file 2 and Additional file 6: Figure S4.

### Population structure of *H. parasuis*

#### Identification of two divergent clades of H. parasuis

**Figure 2:**
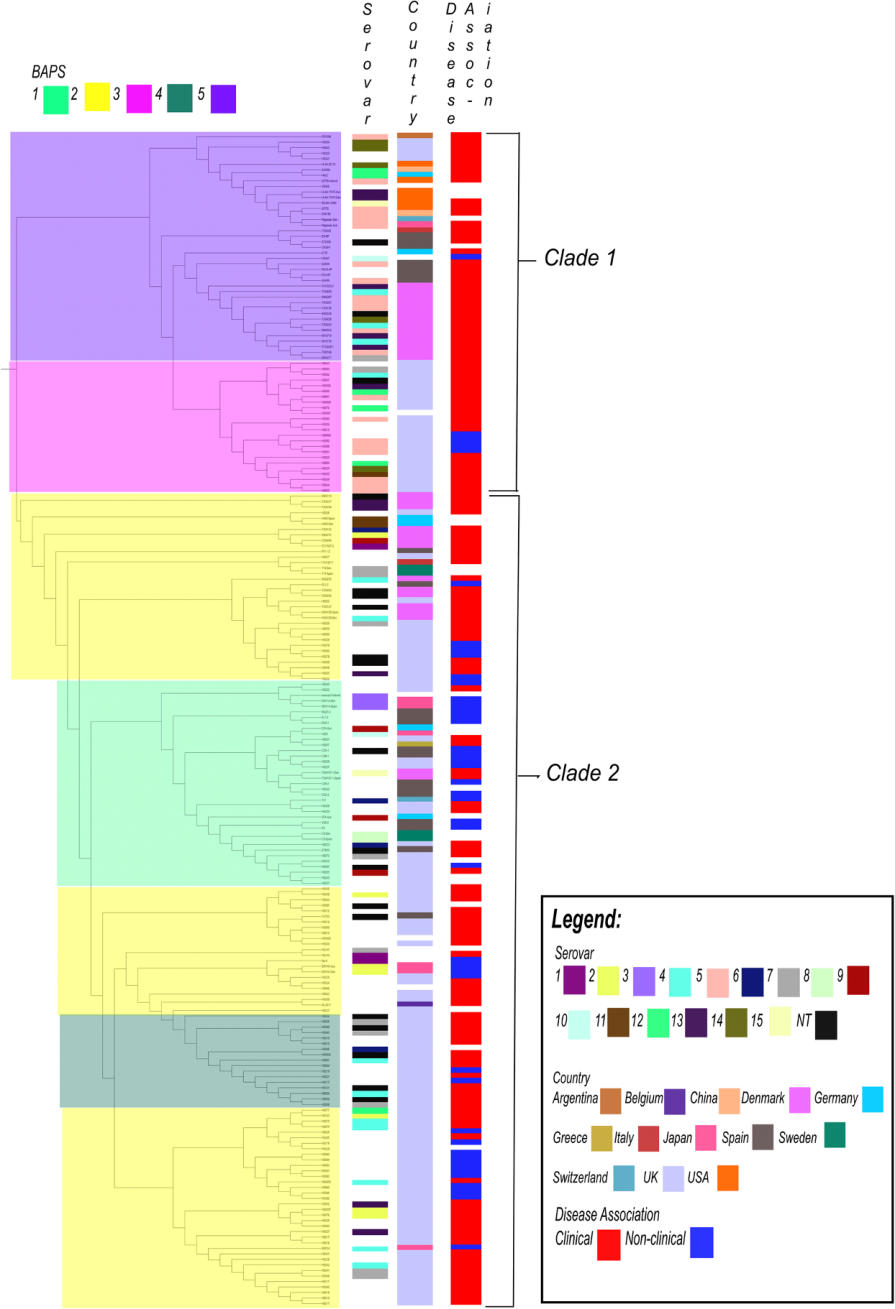
**Core genome Neighbor-joining tree (with areas of recombination included) of** ***H. parasuis*** **(500 bootstraps).** Trees are overlaid with the populations from the Bayesian analysis of population structure (BAPS), which represent isolates with similar rates of homologous recombination. Further metadata including disease association, serovar and country of origin are also shown. BAPS populations explain the separation of the isolates on the tree into two main clades.

**Figure 3:**
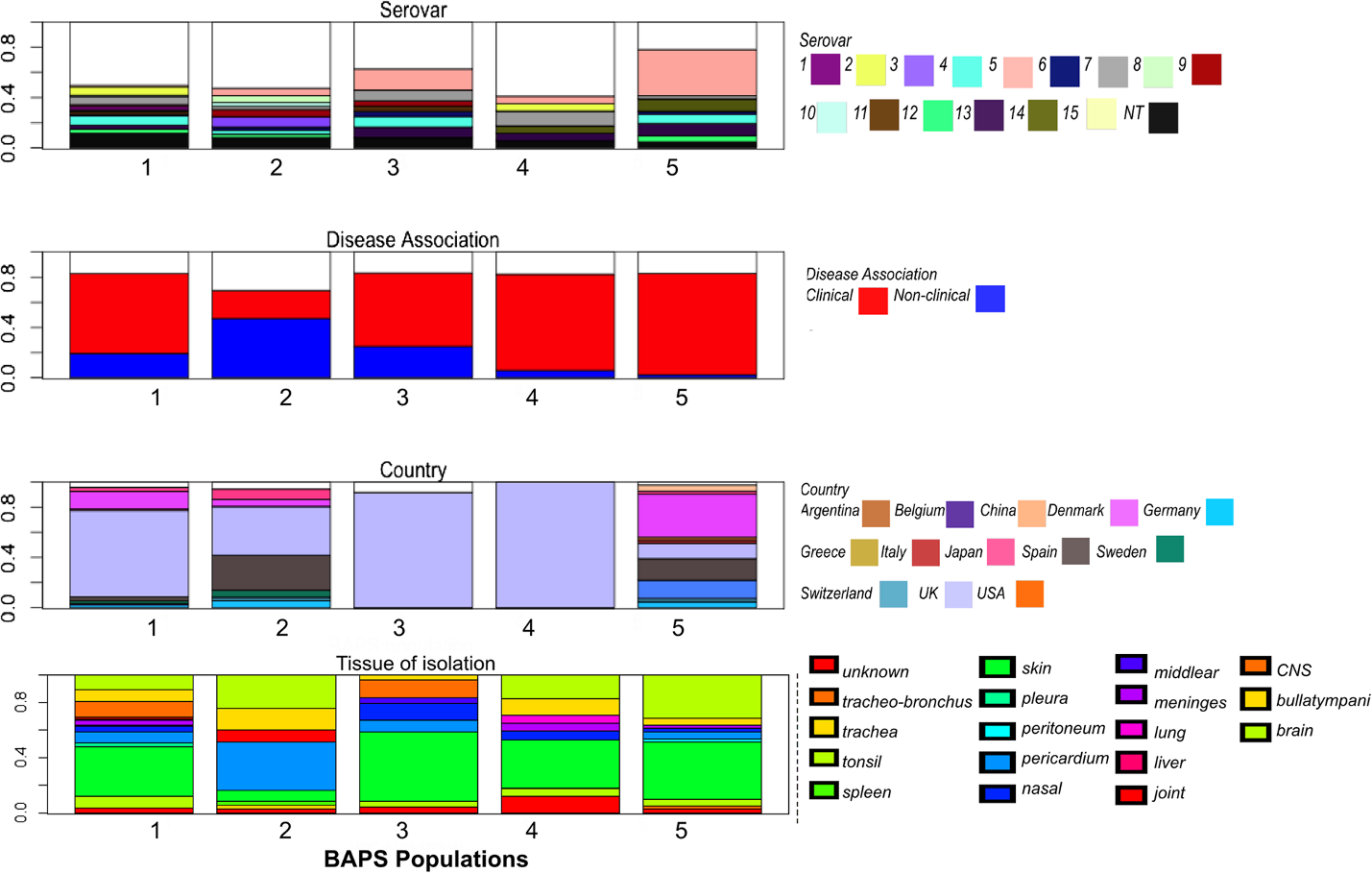
**The BAPS populations defined using Bayesian analysis of population structure represent isolates with similar rates of homologous recombination.** The BAPS populations have been compared to serovar, disease association and country of origin. Some similarity in the BAPS populations 3 and 5 can be seen based on serovar distribution. Very little difference can be seen between BAPS population when disease association is considered. UK isolates are overrepresented in BAPS populations 1, 3 and 4, while 2 and 5 are of mixed geographic origin.

Analyses of the population structure of this isolate collection was performed using Bayesian analysis of population structure (BAPS) [62, 63] on the core genome. Five BAPS populations were identified from these analyses, these represented groups within the overall population that share similar SNPs and indicate a common ancestry with similar amounts of admixture. The BAPS plot shown in Additional file 7: Figure S5 demonstrates the within-cluster and between-cluster variation for the five BAPS populations based on the core genome. While the partitioning of population structure in other bacteria has been reported before [64–66], further analysis of the causes of the partitioning usually leads to an explanation dependent on ecological separation e.g. due to geographical separation or restriction to different hosts or tissues [56, 66, 67]. *H. parasuis* has only been identified in pigs and wild boar, and the route of infection is via the nose and the upper respiratory tract and so are all able to inhabit the same niche [3, 68]. Furthermore, the global nature of the pig industry means that import and export of stock occurs worldwide and so we did not expect that geographical separation would have much influence on the population structure. We began our investigation into the population structure by checking these potential ecological barriers by comparing our clinical metadata (country, disease association and serovar) to the phylogenetic tree of the *H. parasuis* core genome built from the concatenated core genes (Figure 2) [69, 70]. The topology of the phylogeny shows the segregation of the tree into two clades, which fits with the separation of the BAPS populations with BAPS populations 3 and 5 found in clade 1 and groups 1, 2 and 4 in clade 2. We also investigated the correlation between the BAPS populations (level 1) and the clinical metadata (Figure 3). When both the core genome and BAPS populations (Figures 2 and 3) were compared to the metadata, some separation of the serovars can be seen between the two clades, with serovars 5, 12 and 13 identified predominantly in clade 1. Serovar 4 is the only serovar to be found in equal proportions in both the clades. Serovar 7 is mostly found in clade 2. For the remainder of the serovars, the number of isolates was too low to be able to restrict them to one clade. In comparison, no influence of disease association on population structure was observed, as the clinical isolates were found throughout the tree and the BAPS populations (Figure 2). This is contradictory to the population grouping predicted by the multi-locus sequence typing (MLST) scheme [27], which showed six main subgroups on the MLST phylogeny; one predominantly clinical, one mostly non-clinical and four mixed clades. Analysis of the individual BAPS populations in Figure 3, showed that BAPS populations 4 and 5 contained very few non-clinical isolates (5.9% and 2.4% respectively). Some geographic structure can be seen in Figure 2, with a greater proportion of the UK isolates (n = 121) in clade 2 which is likely to be due to the large proportion of UK isolates in the collection. With the global nature of the pig market it is not unexpected that isolates from different geographic locations or serovars would be found in each of the BAPS populations. Indeed, populations 2 and 5 are very mixed; indicating only limited separation by a geographic barrier. The BAPS populations are also very mixed by tissue of isolation and so no tissue tropism can be seen based on the population structure. Of the categories of metadata, only serovar shows some association with the BAPS populations and the differences in recombination. Therefore we conducted a second level BAPS analysis which split the current five BAPS populations into more defined populations; however the second level BAPS analysis still did not explain the population structure (Additional file 2 and Additional file 8: Figure S6).

#### High rates of recombination were identified in H. parasuis

Analysis of the regions undergoing recombination in the core genome was performed using Bayesian recombination tracker (BratNextGen) [71] on the two separate clades present in Figure 2. We decided to perform separate analyses because the long-branch separating clades 1 and 2 would reduce the statistical power to detect recombination in either clade in a joint analysis. The recombination analysis was also performed using gubbins [59, 72], which was performed on the alignment for all isolates. We were able to calculate the recombination rate relative to the mutation rate (r/m) or homologous recombination rate for the individual BAPS populations using the output from gubbins; BAPS 1 had the highest r/m of 660, followed by BAPS 5 (395), BAPS 3 (268), BAPS 4 (144) and finally BAPS 2 (87.9). However, each BAPS population contained isolates with a range of recombination rates, with multiple isolates with a r/m of 0. When the second level BAPS populations were considered, some clusters of isolates with r/m of 0 were identified but the majority of these isolates were still separated between the 18 groups identified in the second level analysis. Even with these isolates with low estimates of recombination rates, the r/m for each BAPS population is very high compared to published recombination rates in bacteria and is far higher than previously estimated for *H. parasuis*. One previous study calculated the r/m for *H. parasuis* based on the genes involved in the MLST scheme [73], and identified higher rates for the clinical isolates (r/m 5.5) compared with the non-clinical isolates (r/m 2.7). Our estimates for the rates of homologous recombination in *H. parasuis* far exceed the estimates based on the MLST housekeeping genes. This may be due to purifying selection that is likely to be affecting housekeeping genes and so the estimate based on the core genome, which contains far more genetic information, should give a more informative estimate of recombination across the genome [59, 73].

The values of r/m of the two clades of *H. parasuis* estimated using both recombination analysis methods were within an order of magnitude. The r/m for the first clade was 663 and 707.7, while the r/m for the second clade was estimated at 892.2 and 575.1, using gubbins and BratNextGen respectively. While both methods yielded comparable estimates of homologous recombination rates, the diversity in this *H. parasuis* population is greater than is usually included in the analysis using gubbins and so care should be taken when interpreting the results. Gubbins was designed to identify recombinations from distant sources within very clonal populations and so these r/ms may even be an under-estimation. In comparison BratNextGen identifies recombination by finding patterns of shared SNPs and so should perform better on this diverse dataset, but did require the analysis of the clades separately.

#### Search for the cause of the population partition in H. parasuis

**Figure 4:**
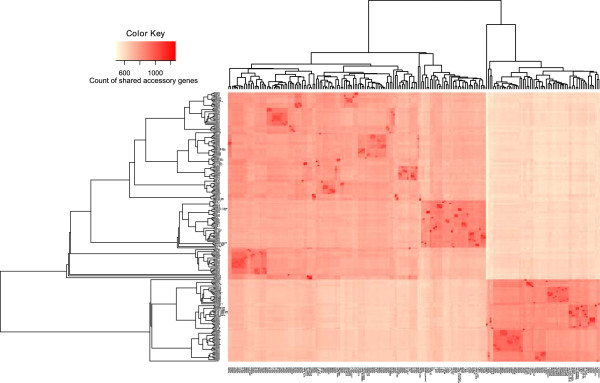
**Heat-map of the shared accessory genes between strains.** The plot is ordered by the phylogeny based on the SNPs within the core genome (rows). While the columns are ordered based on the similarity in the presence and absence pattern of accessory genes between isolates, which is represented by a dendrogram along the top of the heat-map. A clear separation can be seen between the clades, and it appears that both the phylogeny and the dendrogram split the population into the two clades, suggesting little recombination occurs between the two clades, but there is recombination within them both.

Evidence of different restriction modification systems modulating homologous recombination has been associated with clades of *Neisseria meningitidis* but evidence of recombination between the clades was still found in this bacterium [74]. Several methods were used to try to elucidate the differences between the BAPS populations and the two clades of *H. parasuis* using genome wide association methods (GWAS), applied to both the core and the accessory genomes. As we have seen some separation of serovars between these clades (Figure 2), it is possible that differences in the expression patterns of the capsule between serovars may present a recombination barrier [72]. However, recombination within the clades is still occurring between isolates of different serovars. The differences between the clades based on the accessory genome and the phylogeny (Figure 4) was visualised by creating heat-maps of the shared accessory genes between isolates. The heat-map is ordered based on the phylogeny which was built using the SNPs from the core genome (rows), and a dendrogram built based on the similarity in the accessory genome or shared genes (columns). Clear separation can be seen in the accessory genome between the two clades, shown in Figure 4, with only an additional 500–600 accessory genes shared between the two clades aside from the 1049 genes from the core genome.

Additionally, the separation of two clades based on the accessory genome strongly suggests that horizontal transfer occurs in both clades, but that transfer of genetic material is restricted between these groups. This plot allowed us to identify several areas of high similarity between strains, for example isolates HS0001, HS0006, HS0008, HS0009, HS043, HS047 and HS061 share five intact phage regions that were previously identified in *Aeromonas* [GenBank: NC_019541]*, Burkholderia* [Genbank:NC_005882]*, Mannheimia* [Genbank:NC_008201]*, Salmonella* [Genbank:NC_010495] and *Shigella* [Genbank:NC_022749] based on their hits from the PHAST database. Furthermore, a plasmid with similarity to one isolated from *Haemophilus influenzae* (ICEhin1056) was identified in nine isolates using ICEberg. In addition, we were able to identify an integrated antibiotic resistance gene element with greater than 99% identity to one identified in *Vibrio cholera* [GenBank: AB114188]*,* in the two strains possessing multiple antibiotic resistance genes (F9 and HS033).

A BLAST analysis of all known *H. parasuis* genes involved in recombination, competence and restriction modification genes was performed using the isolate collection, but the only difference found was an overrepresentation of transposases in clade 1 compared to clade 2. Experimental data on the natural competence of isolates in this collection are sparse. We have tested a small number of isolates to date, including strains 174 (BAPS 1), HS061 (BAPS 3) and HS069 (BAPS 3), which have been classed as naturally transformable, while 29755 is not transformable (BAPS 5). The presence of naturally competent isolates in both clades suggests that the partition between the two clades is not due to differences in competency genes. Therefore, to investigate this separation between the clades, we performed a GWAS using discriminant analysis of principal components (DAPC) on the BAPS populations. This allowed us to identify genotypes (either SNPs or gene presence/absence) associated with population structure (either based on the two clades or BAPS populations) within our isolate collection. We also used generalised linear modelling (glm) to test if these genotypes were significantly different between the BAPS populations (p < 0.05), whether SNP-based or based on gene presence/absence. We varied the quantities of PCA eigen-values (between 60-90%) included in the DAPC as a cross-validation technique to allow for comparison of the candidates that correlated with population structure.

The DAPC analysis of the core genome showed a clear separation between the BAPS populations (Additional file 9: Figure S7a), and this is also evident between the two clades (Additional file 9: Figure S7b). Similarly, separation of the BAPS populations and clades can be seen based on the accessory genome (Additional file 9: Figure S7c and d) although to a lesser extent than from the core genome. Due to the large separation between the BAPS populations and the clades, a large number of genes and SNPs were identified from this analysis. Even with the top 10% of the loadings from the DAPC (representing the importance of each gene or SNP in the analysis); the numbers of genotypes identified from the DAPC cross-validation was greater than 30 for both SNPs and the accessory genome. The shortlist of 33 SNPs identified from the DAPC of the core genome is shown in Additional file 10: Table S2. However, only 6 SNPs were identified as significantly different between BAPS populations or the two clades and they did not result in a predicted amino acid change in the encoded protein (p < 0.10). Two SNPs were identified within the Holliday junction ATP-dependent DNA helicase (RuvB) and resulted in amino acid changes. It is interesting to speculate that these SNPs may explain the difference in recombination rates between the two clades.

The shortlist of 39 genes identified from the DAPC of the accessory genome is shown in Additional file 11: Table S3, with only four genes identified as significantly different between BAPS populations (p < 0.05). Of the genes in the shortlist, there are several genes encoding outer membrane proteins and potential capsule polysaccharide proteins, two transcriptional regulators, and genes of unknown function (n = 10) that may potentially play a role in the uptake or exchange of DNA. PFAM analysis [75] of the proteins of unknown function (Additional file 11: Table S3) identified domains involved in transporter-associated domains, a helix-turn-helix domain and a lipoprotein domain, and three domains of unknown function, which are not obviously associated with the separation of the two clades.

Based on the heat-map (Figure 4) and the DAPC plots (Additional file 9: Figure S7), we suggest that there are a greater number of genes that are contributing to this separation but they are not found exclusively in particular BAPS populations or clades. This analysis has helped to understand some of the differences between the groups, and the SNPs within the Holliday junction helicase are worth investigating further. The pronounced difference between the two clades of *H. parasuis* warrants further investigation to help us to understand the separation of the two lineages in *H. parasuis* at both a genomic and functional level and their potential involvement in the barrier to recombination between these two clades.

### Genomic differences between serovars

Historically, serotyping has been the main basis of subcategorising *H. parasuis*, and the currently available vaccines are serovar-specific. Although surveillance is not carried out routinely in many countries, it appears that the epidemiological profile of the serovars has been fairly stable throughout the last 30 years [17, 18, 76]. Multiple sets of reference strains of *H. parasuis* are currently in use around the world, and so we have sequenced these strains and confirmed that those with the same name are identical, with the exception of D74 (serovar 9). The two strains labelled D74 shared only 1713 genes of 2123 genes (average number of genes predicted per isolate) and differ by 58 SNPs in the core genome, which places them within the same clade but they belong to different BAPS populations. Reference strains that differed between sets were found to possess the same capsule locus but were otherwise distantly related based on the core and accessory genome. We have found a high association between the capsule locus types and the serotyped isolate collection and propose that the capsule locus is the main determinant of serovar as has been previously assumed [16] (for details see Additional file 2 and Additional file 12: Figure S8). This association was also evident from capsule locus synteny (Additional file 5: Figure S3) when the organisation of the capsule locus of the reference strains was compared by BLASTn to the remainder of the isolate collection [16]. We have also examined the serotyped isolate collection using DAPC and glm to try to identify genes involved in serovar designation, this being detailed in Additional file 2 and Additional file 13: Figure S9. Briefly, based on the prevalent serovars in the isolate collection we were able to identify twenty-three potential serovar-specific genes, five of these candidates were previously identified capsule genes, three phage genes, a transposase, a filamentous haemagglutinin and 13 have unknown functions. This is the first time that a GWAS method has been used to try understand an existing typing scheme. This analysis was based on a relatively small subset of strains when considering the number of isolates of each individual serovar, and so with a greater number of the less prevalent serovars, these methods have a higher likelihood of finding further serovar-specific gene markers. This would allow for the refinement of the typing scheme to a molecular based test, which could have higher turn-around time, better sensitivity and specificity, and maximal reproducibility between laboratories through not depending on antibody reagents and the technical prowess of the laboratory worker.

### Genomic differences in the pan-genome by disease association

#### Putative virulence factors are mostly found in the core genome

The majority of studies proposing virulence factors for this bacterium have focused on a subset of the most prevalent serovars or on a small number of isolates, and so we investigated the distribution of these suggested virulence factors within our more diverse isolate collection. A survey of the literature produced a list of 189 putative virulence factors that were retrievable from NCBI as either a nucleotide or protein sequence (Additional file 14: Table S4) [28–31, 37, 38, 44, 77–79]. This included the *vtaA* genes, which are some of the best characterized virulence markers for *H. parasuis* identified to date [32, 33], but which were found at low frequency in this isolate collection. This may be due to the repetitive nature of the nucleotide sequences of trimeric auto-transporters, which means that these may not have assembled well in our draft genome data. Instead we used the translocator and leader sequences of these genes and identified the group 1, 2 and 3 translocator domains in all isolates. Of these proposed virulence factors, 123 were present at greater than 80% identity in all of our isolates and so were present in the core genome. There is the possibility that variation within these genes may contribute to differences in virulence but this analysis was beyond the scope of this study. The distribution of the putative virulence factors from the accessory genome was examined with respect to disease association (clinical or non-clinical) and disease phenotypes (systemic, respiratory and non-clinical) by the calculation of their relative proportions. Five virulence factors were over-represented in the non-clinical isolates (*cjrA,* K756_04470, K756_03455, *yfeA*, and the leader sequence for non-virulent *vtaA*) and under-represented in the clinical isolates. Conversely, 10 virulence factors were over-represented in the clinical isolates (*bioB, cirA, fimB, hicA, hicB, hhdB,* F357_gp34, K756_02745, K756_06920, and leader sequence of *vtaA* s denoted VIR1). When considering the disease phenotypes, no virulence factors were over-represented in only the respiratory population and only *hsdR* and F357_gp36 were over-represented in systemic isolates. We also looked into the differences in genome size and number of genes between the disease associated isolates, as a proxy for evidence of reductive evolution [80, 81]. No evidence of reductive evolution was found based on our isolate collection; details can be found in Additional file 2 and Additional file 15.

#### A high number of genes from the accessory genome were linked to differences in clinical phenotype

**Figure 5:**
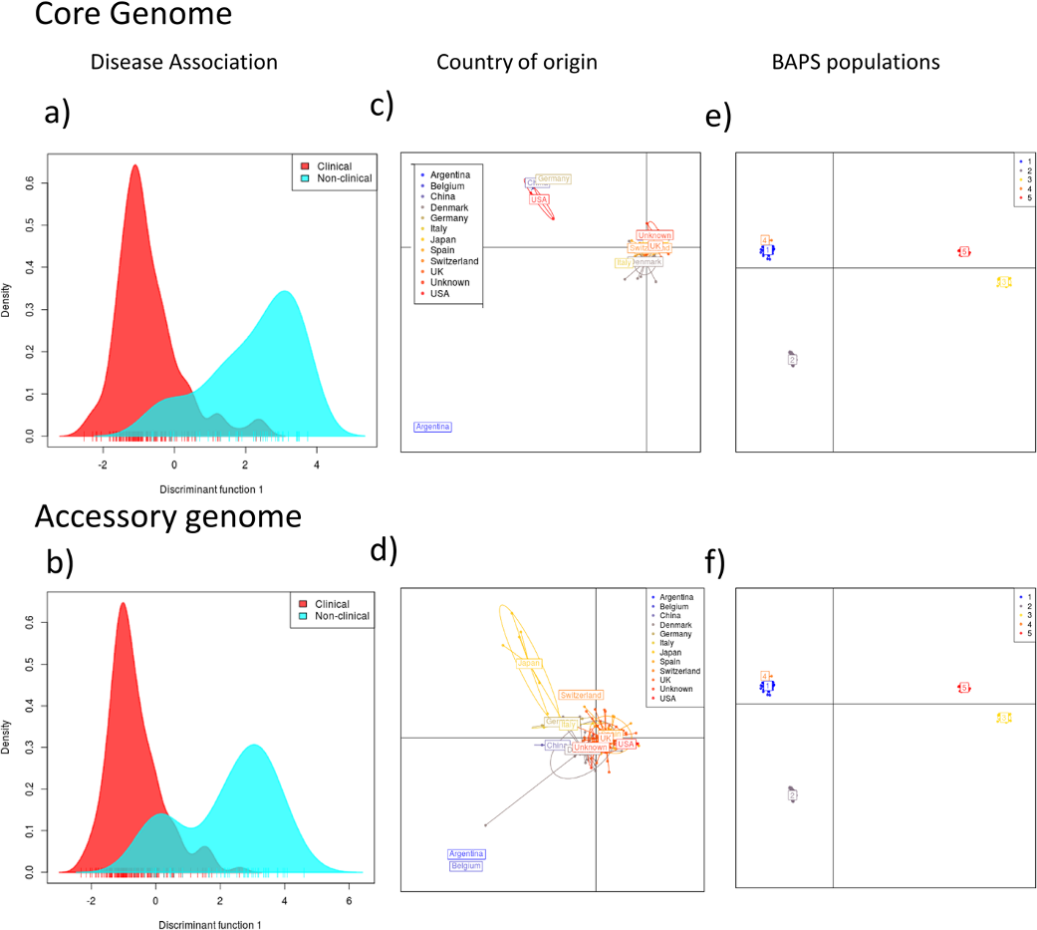
**Discriminant analysis of principal components applied to clinical strains of** ***H. parasuis*** **by disease categories.** 80% eigenvalues were retained for the PCA and all eigenvalues were retained for the discriminant analysis. Plots **a** and **b** show the first axis of the discriminant function while **c**, **d**, **e** and **f** show the first two axes. Separation along the axes suggests that genetic differences are present between the phenotypic groups of clinical and non-clinical isolates that are being compared; however the presence of overlap shows that some strains are intermediates. Plots c and d show that geographic origin, do not show much separation of the isolates by geography, those that have separated are only represented by a couple of isolates. On the other hand, the discriminant function based on the BAPS populations shows a lot of separation and so the population structure does have an influence on these isolates genetic content.

While we were able to find some associations between existing putative virulence factors for *H. parasuis* and clinical phenotype*,* there is also a large amount of data from the pan-genome that could yield new important SNPs or genes that are associated with pathogenicity. Therefore, we used the methods of DAPC and glm to assess the relationship between the pan-genome and virulence based on the clinical metadata. From this analysis we have identified both SNPs and genes that allow us to separate the isolate collection into disease categories. All of the candidates were added into the glm with clinical disease as the factor in the model, and those that were significantly different between the clinical and non-clinical isolates (p < 0.05) were considered as potential markers for virulence. We also performed analyses of disease phenotypes for both the core and the accessory genome, but very little difference between the respiratory and systemic isolates was identified using DAPC (data not shown). This occurred whether isolates from the lung with signs of systemic disease were classed as either respiratory or systemic strains. This suggests that the difference between respiratory or systemic outcome of disease may be dependent on additional host factors such as welfare and host immune response rather than genetic differences between the isolates or particular tissue tropisms. The discriminant function of the disease association is shown in Figures 5a and b for the core and accessory genomes respectively (with 80% of the PCA eigen-values retained). This shows separation between the clinical and non-clinical isolates to a similar extent for both the core and the accessory genome. Some overlap was seen between these categories (Figures 5a and b), but this is not unexpected as some isolates may be capable of causing disease but were not doing so at the time of isolation. We also considered the influence of geography and the separation of the strains into populations with different recombination rates (based on the BAPS populations) on the pan-genome and saw that both showed separation in the discriminant functions of the core and accessory genomes (Figures 5c, d, e and f).

When we consider the candidate markers for virulence from the DAPC and the glm, a greater number of candidates were significantly different between the clinical and non-clinical isolates from the glm of the accessory genome (48 candidates from the accessory genome, which are listed in Table 1) over the core (12 SNPs in the core genome). Of the SNPS identified as being statistically significant, seven resulted in predicted amino acid changes. However, only four predicted the change of amino acid type, which may influence the structure of the protein. For example, two SNPs identified in a long chain fatty acid CoA ligase (1186G/T) and in a transcriptional regulatory protein (1384G/T), which would result in an A/S amino acid change and a change from hydrophobic to polar. A SNP in a fatty acid oxidation complex subunit alpha (1789A/G) was predicted changing the amino acid from hydrophobic to polar and finally a aromatic to hydrophobic change at holo-acyl carrier protein synthase (435C/A). A gene involved in fatty acid degradation (*fadA*) was previously suggested as having a role in virulence in *H. parasuis*[34]*.* Furthermore, the association of fatty acid metabolism and virulence has been previously identified in *Mycobacterium tuberculosis*[82]*.* Of the genes yielded from the accessory genome analysis nine were putative virulence factors from the previous analysis of published virulence factors (Table 1). From the DAPC pathogenicity candidates we identified genes encoding four adhesins, ABC transporters, an antirepressor, an aspartokinase, two cytolethal distending toxins, enoyl-CoA hydratase/carnithine racemase-like protein, ferric uptake proteins, glycerol uptake facilitator protein, a hemolysin transporter, a methylase, an N-acetylmuramoyl-L-alanine amidase, a nucleoside symporter, an outer membrane protein, four phage proteins, an RNA helicase, a putative serine protease, a *tonB* dependent receptor, another toxin (PezT), a transcriptional activator, two transposases, a xylose import ATP-binding protein and nineteen proteins of unknown function. The genes of unknown function were run through the PFAM database (Finn et al. [75]) to try to elucidate any further details about their function, but only three had any hits to known domains, one with a phage domain (evalue 7 × 10^-5^), one with an ABC transporter and membrane domains (evalue 1 × 10^-48^) and another with a YadA adhesin domain (evalue 1.5 × 10^-4^).

**Table 1:** **Significantly different genes identified from H. parasuis between clinical and non-clinical isolates using DAPC**

Predicted function of gene	Number of genes	Existing virulence factor for ***H. parasuis***
Adhesins, *yadA* family	4	Yes
antirepressor	1	
Aspartokinase *lysC*	1	
ATPdependent RNA helicase *hrpA*	1	
cytolethal distending toxin protein A	2	Yes
cytolethal distending toxin protein B	1	Yes
DNA binding domain, excisionase family	1	
enoyl-CoA hydratase/carnithine racemase-like protein	1	
Ferric hydroxamate uptake	1	
Gene 25like lysozyme	1	
glycerol uptake facilitator protein	1	
Modification methylase *hpaII*	1	
MuF Haemophilus phage SuMu	1	
N-acetylmuramoyl-L-alanine amidase	1	
phage tail sheath family protein	1	
putative 3-phenylpropionic acid transporter	1	
Putative NADH flavin reductase/short chain dehydrogenase	1	
putative outer membrane usher protein *htrE*	1	
putative serine protease	1	Yes
tonB-dependent receptor plug domain protein	1	Yes
Trans-2,3-dihydro-3-hydroxyanthranilate isomerase	1	
Transcriptional activator *prtR*	1	
transposase	1	
UDP3O [3hydroxymyristoyl] glucosamine N-acyltransferase	1	
unknown function	19	
Xylose import ATPbinding protein *xylG*	1	

All genes were identified as significant using generalised linear modelling of different DAPC iterations using between 60-90% of the principal components (p < 0.05). The phenotypic data used in the model was whether isolates were clinical or non-clinical compared to the presence or absence of the genes.

These genes are in the same families as other common virulence determinants in other better-studied pathogens. For example, adhesins are commonly regarded as virulence factors as they allow adhesion to the host cell, and depending on their specificity may be required for the progression of disease in particular tissues [83]. While cytolethal distending toxins have been previously studied in *H. parasuis*[35, 84]*,* there appear to be multiple, divergent copies within the isolate collection. Variation in these *cdt* genes has previously been found between different strains of *E. coli* at as little at 39% amino acid identity, and so the presence of multiple copies of this family of toxins may impact the virulence of isolates of *H. parasuis*[85]. In addition, glycerol uptake facilitators have been implicated in virulence and growth in *Mycoplasma pneumoniae*[86]*;* N-acetylmuramoyl-L-alanine amidases play a role in the conversion of the *Streptococcus mitis* autolysin [87]; serine proteases are regarded as virulence factors in enterohemorrhagic *E. coli*[88] and *Streptococcus pneumoniae*[89] and the *tonB*-dependent receptors are involved in iron acquisition, which is important in pathogens such as *Neisseria meningitidis*[90] and *Vibrio cholera*[91]*.* In summary, the DAPC and glm analyses of the isolate collection have yielded a short-list of new putative virulence factors for further investigation in this pathogen and their use as epidemiological markers will be considered further.

## Conclusion

We have investigated the composition of the pan-genome of the major pig pathogen *H. parasuis* with respect to detailed metadata in order to investigate associations between genotype and phenotype. We have found that this is a diverse bacterial species, comprised of two main lineages, exhibiting very high rates of recombination, which appears to be restricted within one or other of the two clades. Using DAPC as the GWAS method, we were able to identify SNPs within a Holliday junction ATP-dependent DNA helicase (RuvB) that may be involved in the partitioning of this species into two clades. We have found genetic differences with regard to serovar based on the capsule loci and further potential serovar-specific genes from the accessory genome, which is the first time that a GWAS method has been used to try to refine an existing typing method. We have also identified genetic differences in the clinical and non-clinical isolates from 48 genes, nine of which were also identified by DAPC and glm analyses. The discriminant power of the accessory genome over the core genome provides a better list of candidate virulence genes that will be invaluable for investigating the pathogenesis of *H. parasuis* and how it causes Glässer’s disease. The accessory genome is therefore an important source for understanding the pathogenesis of diverse species of bacteria, particularly in combination with detailed metadata. These methods are applicable to any pathogen where detailed clinical metadata can be obtained and analysed in combination with next generation sequencing, which could identify genotypes (both SNPs and genes) that may be useful in the generation of new molecular diagnostic and vaccine candidates that can aid surveillance and prevention of disease.

## Methods

### Isolate collection

The table describing the isolate collection and metadata (including accession numbers for the isolates) can be found in Additional file 1: Table S1. This collection includes the reference strains currently used in the serotyping scheme and three published genomes of 29755, SH0165 and ZJ0906 in the analyses [40, 41, 60]. There are currently multiple isolates used as reference strains for the same serovar in different serotyping laboratories, and so we have sequenced 27 reference strains from three different laboratories [61]. UK isolates were obtained from the Animal Health Veterinary Laboratories Agency (AHVLA) and were identified as *H. parasuis* on the basis of minimum biochemical tests for differentiating *H. parasuis* (haemolysis, CAMP, urease, indole and catalase) and purified in the UK [92]. The remainder of the isolate collection was provided by Centre de Recerca en Sanitat Animal (Spain), National Veterinary Institute, Technical University of Denmark and Patrick Blackall (University of Queensland, Australia). Metadata for these isolates includes: veterinary investigation diagnostic analysis codes used in the UK (VIDA), tissue origin of isolate, age of pig, ante and post-mortem findings and for a subset of isolates serotyping results (117/212 isolates). Serotyping for the UK isolates was performed by indirect haemagglutination [11, 93] by Innovative Veterinary Diagnostics, Germany. Historical isolates obtained with serotyping information, including the reference strains, were serotyped by both gel diffusion and indirect haemagglutination by a variety of laboratories [11, 61, 93].

Metadata for the strains was used to classify the isolates with clear diagnostic information (n = 186) and were separated into clinical (n = 143) and non-clinical isolates (n = 43). The clinical isolates were then separated further into disease phenotypes of respiratory (n = 76) and systemic (n = 49), with 18 isolates that we were not able to classify as respiratory or systemic based on the diagnostic information and which were excluded from the disease phenotype analysis.

Basic descriptive epidemiological analyses of this isolate collection were performed including assessment of the relationship between serovar and virulence in our dataset, as this is a commonly assumed relationship. Of our dataset, 20 isolates (14.5%) with an assigned serovar also had cross-reactions; we have chosen to ignore the cross-reactions for the statistical analysis as it reduces the number of isolates in each group, reducing the power of the statistical analyses.

### Genome sequencing and assembly

Isolates were sequenced using published methods [16]. Draft genome sequences were assembled using a custom-made bioinformatics pipeline, detailed in Additional file 2. Assembly statistics were checked to see if any isolates required repeat sequencing. The final assemblies ranged in size between 2,192,774 and 2,254,362 bp, with 39.48-39.94% average G + C content, which was comparable to the publicly available draft and complete genomes of *H. parasuis*[40, 41].

### Construction of the pan-genome

The pan-genome of *H. parasuis* was investigated using OrthoMCL [94]. The amino acid sequences of predicted proteins were obtained through prodigal analysis of the isolates [95], and were used to represent the coding sequences (CDSs) of all of the isolates. The homology groups represent the unique proteins amongst the pooled proteins predicted from all isolates, which can then be used to infer the pan-genome. The MCL parameters were varied to create the optimum core genome, using the lowest inflation parameter possible to get the highest number of homology groups that can be identified in every isolate, maximising the core genome. The final inflation parameter used was 2.0, and while this low value may produce more false positives, we also added a series of manual checks on the homology groups after the OrthoMCL analyses to further ensure the accuracy of the pan-genome. This was necessary because of the large number of draft genome sequences used and allowed us to account for sequencing errors and pseudogenes. It also allowed us to minimise the number of homology groups with duplicates from the same isolate. For details of the homology group checks that have been performed see the Additional file 2. Automated genome annotation was performed using Prokka using the rfam option [96].

Custom python scripts were used to recreate the MCL table of the homology groups based on the altered groups and to convert the homology groups to a presence and absence matrix across the isolates. Homology groups found in every isolate were treated as the core genome and a Fasta file of the gene sequences from all isolates was produced for each homology group. These Fasta files were aligned to confirm the core genome, enabling exclusion of any genes that aligned with less than 80% identity. The remainder of the homology groups were regarded as the accessory genome. The alignments of the core genes were combined to create a concatenated alignment of the core genome. MEGA was used to create a Neighbor-joining core genome tree using 500 bootstraps [97]. A subset of individual core gene trees were also built and bootstrapped (100 times) using MEGA to facilitate the comparison of the relationship between the isolates across the genome as well as for the concatenated alignment [97]. COG information for the homology groups was obtained using the protocol detailed in Additional file 2. Heat-maps of the shared accessory between isolates, were created using R and the R libraries vegan [98], gplots [99] and Heatplus [100].

### Recombination analyses of the core genome

Recombination and population genetic analyses of the concatenated core genome was performed using Bayesian analysis of population structure (BAPS) [62, 63] and Bayesian Recombination Tracker (BratNextGen) [71]. BAPS clusters were estimated using 10 independent runs of the stochastic optimization algorithm with the *a priori* upper bound of the number of clusters varying over the interval 50–100 across the runs and using two levels of hierarchical analysis. All runs converged to the same posterior estimate of the population structure with 5 and 18 clusters at the nested first and second levels of hierarchy, respectively. All clusters in the two estimates were significantly supported when compared against alternative partitions (posterior probability of any cluster at least 100-fold higher than for the alternative). The BratNextGen estimation of genomic locations of recombination was performed with the hidden Markov model hyperparameter alpha set to 1 and using 20 iterations of the estimation algorithm, which was assessed to be sufficient since changes in the hidden Markov model parameters were already negligible over the last 60% of the iterations. The significance of a recombining region was determined using a permutation test with 100 permutations executed in parallel on a cluster with a threshold of 5% to conclude significance for each region. The ratio of the recombination rate relative to the mutation rate (r/m) was calculated using custom scripts based on the BratNextGen output [56, 65]. This procedure was used for the two separate clades of *H. parasuis.* Recombination analysis was also performed using gubbins as detailed in [59]. As recombination can heavily influence the population structure [101, 102], an alignment with the areas of recombination masked as missing data was attempted create a recombination-free core genome. However, only 425 bp were retained and so a recombination free core genome phylogeny could not be computed.

### Synteny analysis of the pan-genome

Synteny of the homology groups based on the published isolates was examined by using the contig numbering within the accession numbers of the genes (as those created by prodigal include the contig number as well as the position of the CDS). Firstly the order of genes within all of the individual contigs (largest to smallest) of all isolates was established, followed by finding the corresponding homology groups. We used the complete genome SH0165 as the reference for the syntenic pan-genome [40]. If a gene was located next to one already included in the pan-genome then it was inserted into the pan-genome relative to this position. This was converted to an ordered matrix of homology groups within the contigs using custom python and R scripts. The functions of the homology groups were identified by using BLASTp searches of the individual homology groups against the NCBI nr database (updated December 2013). This process was repeated using the just the capsule genes to establish the synteny of the capsule loci (Additional file 16: Figure S10), based on the organisation of the capsule loci of the 15 reference strains [16].

The %G + C content average of homology groups was calculated using a custom python script. Mobile elements were investigated using BLAST analysis of the sequences from the ICEberg database [103] against a database of the isolate collection with no filters or e-values defined and phage were investigated using the PHAST database [104]. This was then combined with predicted Prokka functions and BLASTp hits against nr to build a map of the key features and areas of variation within the pan-genome.

### Statistical analysis of the pan-genome with metadata

We used the R package adegenet to analyse the pan-genome of *H. parasuis*[105, 106]. Discriminant analysis of principal components (DAPC) allowed us to analyse both SNPs of the core genome, and of the accessory genome, based on presence and absence of the homology groups throughout (with pseudogenes excluded). SNPs were extracted from the core genome, and rare alleles (found in <1% of the isolates) were excluded to lessen the influence of sequencing errors on the analyses. Principal component analysis (PCA) was performed on both the core and the accessory genome, and cross-validation was performed by varying numbers of eigen-values (60-90%) in the analysis to capture the variance in the data and all eigen-values from the discriminant analysis were included, after which metadata was chosen for inclusion in the DAPC. DAPC allows the plotting of the discriminant function, which shows the separation between the defined groups (based on the clinical metadata). SNPs or homology groups that contribute most to these differences between groups can be identified and tested for their significance in comparison to the metadata. Step-wise model selection (in both directions) was used to produce a binomial generalised linear model (glm) that allowed the identification of statistically significant SNPs or genes (p < 0.05) in association with the clinical metadata for each round of DAPC [107].

### Proportions of genes of interest in the pan-genome

The presence and absence of genes of interest were assessed using BLAST searches (tBLASTn or BLASTn) of sequences against a BLAST database of the isolate collection. Presence of the gene was defined as above using cutoffs of >80% identity over >80% of the length of the alignment unless otherwise stated, and the proportion of the sequenced isolates which contained each individual gene was determined.

### Association between capsule locus and serovar

The sequences and gene composition of the capsule loci for the 15 reference strains of *H. parasuis* have been published but no comparison to other isolates of the same serovar has been performed. Therefore we compared the capsule loci from isolates of the same serovar in search of an association between the genotype of the capsule loci and the phenotype of serovar. Initial BLAST searches (BLASTn) of the capsule loci (*funA* to *iscR*) of each reference strain, were performed on the BLAST database of all isolates. Isolates that had the same serovar as the reference strain based on a blast match of greater than 90% of the length of the capsule locus and greater than 80% of the identity were considered as a match. Isolates that did not meet these criteria were compared to the capsule loci of the other reference strains using ACT [108]. The reference strain with the best match was based on highest visible identity in ACT across the entirety of the capsule locus. If less than 80% identity at nucleotide level was found to any of the reference capsule loci then we considered there to be no match to existing capsule loci. The isolates with the same capsule loci were then aligned using muscle [109], and the average identity across the capsule locus (*funA* to *iscR*) was calculated using alistat [110].

### Accession numbers

Accession numbers of isolates included in this analysis are available on Genbank [Genbank: ABKM00000000, CP001321, CP005384].

Accession numbers of isolates included in the analysis in the European Nucleotide Archive: [ENA: ERS131963-ERS131990, ERS131992-ERS132006, ERS132009-ERS132069, ERS132072,ERS132073, ERS132075-ERS132090, ERS132095-ERS132117, ERS132119-ERS132123, ERS132125-ER132127, ERS132129, ERS132132-ERS132147, ERS132150-ERS132151, ERS132161, ERS132168, ERS132171, ERS13217-ERS132183, ERS132188, ERS132189, ERS132191-ERS132199, ERS193695-ERS193698, ERS214842-ERS214848 and ERS134375].

### Ethics

Isolates from the UK were obtained from the Animal Health Veterinary Laboratories Agency as part of routine diagnostic investigations upon submitted post mortem material. The isolates provided by CReSA were field isolates obtained from nasal swabs and from clinical diagnostic materials provided by Department of Infectious Diseases of the Veterinary School of the Universitat Autònoma de Barcelona (Spain). Danish isolates originated from pigs showing clinical signs that were submitted to the Laboratory for Swine Diseases (Kjellerup, Denmark) for post-mortem examination. Sources of isolates were anonymised.

## Electronic supplementary material

Additional file 1: Table S1: Is the table describing the *H. parasuis* isolate collection including accession numbers, country of origin, clinical data and serotyping results. (CSV 12 KB)

Additional file 2: **Is the supplemental information** **accompanying this paper including 1) COG functional classification of the homology groups, 2) variable regions in the synteny of the pan-genome, 3) G + C content of the pan-genome, 4) second level BAPS analysis, 5) correlation between the capsule loci and serotyping results, 6) identification of potential serovar-specific markers from the pan-genome, 7) limited evidence of reductive evolution in** ***H. parasuis,*** **8) genome assembly, 9) homology group checks.** (DOCX 46 KB)

Additional file 3: Figure S1: Synteny of the pan-genome created based on the SH0165 complete genome, with black areas showing presence of a gene, and white areas absence. Areas of variation in the synteny with predicted phage genes are highlighted in red, areas with high levels of genes of unknown function in green. Numbers indicate regions of variation. Antibiotic resistance gene locations are represented by orange lines. (PNG 143 KB)

Additional file 4: Figure S2: Synteny of the capsule loci of *Haemophilus parasuis*. Rows represent the isolates from the collection and black diamonds represent the presence of genes. Each isolate was found to possess a capsule locus, isolates have been ordered by their similarity in the pattern. The beginning and end of the locus are conserved but a lot of variation can be seen within the locus. (PDF 733 KB)

Additional file 5: Figure S3: Bayesian Analysis of Population Structure of 212 isolates of *H. parasuis*. Each row in the figure is a strain and each column is a SNP with sites ordered to maximize visual separation of the five distinct clusters. Horizontal black lines are drawn to separate the clusters obtained in the first level of clustering. (PDF 14 KB)

Additional file 6: Figure S4: Discriminant analysis of principal components applied to the BAPS populations and two clades of *H. parasuis* (80% eigenvalues retained for the PCA), all eigenvalues were retained for the discriminant analysis. Plots a and c show the first two axes of the discriminant function while b and d show the first axis only. Separation along the axes suggests that genetic differences are present between the BAPS populations and clades. A large degree of separation can be seen from the both accessory genome and the core genome, and is far more pronounced for the two clades of *H. parasuis*. (PDF 169 KB)

Additional file 7: Figure S5: Comparison of the COG functional groups found in the core and accessory genome of *H. parasuis* found at greater than 1%. Several differences in proportions of COG groups can be seen between the core and the accessory genome. The core genome shows a greater proportion of proteins involved in translation, coenzyme metabolism as well as post translational modification over the accessory genome. A greater proportion of cell wall/membrane biogenesis transcription, replication and repair and cell motility, defence mechanisms as well as proteins of unknown function were identified in the accessory genome. CELLULAR PROCESSES AND SIGNALING -**[D]** Cell cycle control, cell division, chromosome partitioning, **[M]** Cell wall/membrane/envelope biogenesis, [**O]** Post-translational modification, protein turnover, and chaperones, **[T]** Signal transduction mechanisms, **[U]** Intracellular trafficking, secretion, and vesicular transport, **[V]** Defense mechanisms, **[W]** Extracellular structures. INFORMATION STORAGE AND PROCESSING - **[J]** Translation, ribosomal structure and biogenesis, **[K]** Transcription, **[L]** Replication, recombination and repair. METABOLISM- **[C]** Energy production and conversion, **[E]** Amino acid transport and metabolism, **[F]** Nucleotide transport and metabolism, **[G]** Carbohydrate transport and metabolism, **[H]** Coenzyme transport and metabolism, **[I]** Lipid transport and metabolism, **[P]** Inorganic ion transport and metabolism, **[Q]** Secondary metabolites biosynthesis, transport, and catabolism. POORLY CHARACTERIZED - **[R]** General function prediction only, **[S]** Function unknown. If a gene cannot be classified by a singular COG category then multiple categories can be used, e.g. UW represents proteins involved in intracellular trafficking **[U]** and extracellular structures **[W]**. (PNG 115 KB)

Additional file 8: Figure S6: G+C content of the *H. parasuis* pan-genome. Plot a) shows the variation of the G+C content based on the syntenic order of the pan-genome, while plot b) shows a histogram of the variation in all predicted genes. (PNG 317 KB)

Additional file 9: Figure S7.: BAPS populations level 2 comparison to clinical metadata including serovar, disease association and country. Greater separation of serovars (particularly for serovar 5), disease association and geography (for the UK and Denmark) can be seen based on these more refined populations. (PNG 201 KB)

Additional file 10: Table S2.: Lists the single nucleotide polymorphisms identified as significant from the BAPS populations and clades. (XLSX 11 KB)

Additional file 11: Table S3: Is the table detailing the accessory genes that are significantly different between BAPS populations and clades. (XLSX 34 KB)

Additional file 12: Figure S8: Synteny of the capsule loci of *Haemophilus parasuis*. Rows represent the isolates from the collection, coloured diamonds represent the presence of genes. Each isolate was found to possess a capsule locus, isolates have been ordered by their predicted serovar. The beginning and end of the locus are conserved but a lot of variation can be seen within the locus. The pattern based on the predicted serovars does fit with the presence and absence of the genes for the majority of isolates. (PNG 156 KB)

Additional file 13: Figure S9: Discriminant Analysis of Principal Components applied to the core genome and accessory for *H. parasuis* serotyped strains, retaining 80% of the PCA eigenvalues. **A)** Separation of the serovars into two main groups can be seen based on the first two axes of the discriminant function. **B)** Greater separation of the discriminant function of serotyped strains by serovar can be seen from the core in comparison to the accessory. However these serovars have a low number of strains within these groups. (PNG 180 KB)

Additional file 14: Table S4: Is the table listing the putative virulence factors for *H. parasuis* that were investigated in this isolate collection. (CSV 15 KB)

Additional file 15: Table S5: Is the table detailing the association between the capsule loci and serotyping results. (CSV 1 KB)

Additional file 16: Figure S10: Comparison of genome size (based on the number of homology groups and pseudogenes) using box and whisker plots. No difference can be seen in either genome size or number of pseudogenes for disease association, serovar or BAPS populations. (PNG 133 KB)

## Abbreviations

ACT: Artemis comparison tool
AHVLA: Animal health veterinary laboratories agency
BAPS: Bayesian analysis of population structure
BLAST: Basic local alignment search tool
BratNextGen: Bayesian recombination tracker
CAMP: Cyclic adenosine monophosphate
CDS: Coding sequence
COG: Clusters of orthologous groups
DAPC: Discriminant analysis of principal components
glm: Generalised linear model
GWAS: Genome wide association study
*H. parasuis*: *Haemophilus parasuis*
MCL: Markov cluster algorithm
MLST: Multi-locus sequence typing
NCBI: National center for biotechnology information
nr: Non-redundant' database
NT: Non-typeable
PCA: Principal components analysis
PHAST: Phage search tool
PRRS: Porcine reproductive and respiratory syndrome virus
r/m: Recombination rate relative to mutation rate
SNP: Single nucleotide polymorphism
VIDA: Veterinary investigation diagnosis analysis.
