# Supplementary material for: The use of genome wide association methods to investigate pathogenicity, population structure and serovar in Haemophilus parasuis
Source: BMC Genomics. 2014 Dec 24;15:1179. doi: 10.1186/1471-2164-15-1179 (PMC4532294; doi:10.1186/1471-2164-15-1179)
Supplement: Supplementary file 2 — Additional file 2: Is the supplemental information accompanying this paper including 1) COG functional classification of the homology groups, 2) variable regions in the synteny of the pan-genome, 3) G + C content of the pan-genome, 4) second level BAPS analysis, 5) correlation between the capsule loci and serotyping results, 6) identification of potential serovar-specific markers from the pan-genome, 7) limited evidence of reductive evolution in H. parasuis, 8) genome assembly, 9) homology group checks. (DOCX 46 KB) [file 12864_2014_7083_MOESM2_ESM.docx]

**Supplemental Information:**

**S1. COG Functional Classification of the homology groups**

BLAST analysis was performed using an example of each homology group compared to a downloaded database of the COG functional classification database [1, 2] using BLASTp, and results were sorted using an R script to determine the best hit for each group. The matches of homology groups to the COG database were then separated into the core and accessory genome and the proportions of each COG category compared within the pan-genome. Differences were found between the functional composition of the core and the accessory genome (Figure S5). A greater proportion of genes involved in amino acid transport and metabolism (E), nucleotide transport and metabolism (F), coenzyme metabolism (H), translation (J), cell membrane biogenesis (M) and post-translational modification (O) were identified within the core genome compared to the accessory (Figure S5). Genes involved in transcription (K), replication, recombination and repair, cell motility (L), intracellular trafficking, secretion, vesicular transport and extracellular structures (UW), and defence mechanisms (V), as well as genes with a general function prediction only (R), or encoding proteins of unknown function (S), were identified in greater proportion in the accessory genome. Of those genes involved in cell membrane biogenesis (M), 10% were previously identified as capsule genes [3]. The defence mechanism proteins were made up of 52% restriction-modification proteins and 42% multi-drug or antimicrobial resistance proteins.

**S2. Variable regions in the synteny of the pan-genome**

We started with the gene order of the complete genome sequence of SH0165 (Xu et al. 2011) and added genes from the pan-genome whose positions could be estimated. A gene could only be added to the syntenic pan-genome if its position could be estimated based on one of its neighbour’s positions in the pan-genome order. Six variable regions were identified and can be seen in Figure S1. Region 1 encoded a hemolysin operon and contained colicin import genes and many genes of unknown function. For this region the G+C content was consistent with that found in *H. parasuis* but several genes encoded proteins with best BLASTp matches in the NCBI non-redundant protein database (nr) to sequences from *Actinobacillus minor* and *Mannheimia haemolytica.* Region 2 contained several phage genes and genes encoding transposases, as well as a conjugal transfer operon, as well as genes encoding iron-sulphur binding proteins, adhesins, cytolethal distending toxin and proteins of unknown function. Region 3 contained Mu phage genes, genes encoding restriction modification proteins and further adhesins, as well as more proteins of unknown function. Region 4 predominantly consisted of genes encoding proteins of unknown function, but also contained genes encoding a metallo-peptidase and a virulence trimeric autotransporter. Regions 2, 3 and 4 did contain some genes of relatively high G+C content (40-50%) but no hits were found when the encoded proteins were BLASTed against NCBI non-redundant protein database (nr). Region 5 contained another conjugation operon, as well as genes encoding adhesins, transposases and many proteins of unknown function. Region 6 was the only variable region to encode multiple proteins with best hits from other bacterial species in nr, with matches identified from *Actinobacillus pleuropneumoniae* (unknown function [Genbank:WP_005613312.1]), *Gallibacterium anatis* (coproporhyrinogen III oxidase –[Genbank:WP_021461876.1])*, Klebsiella pneumoniae* (coproporhyrinogen III oxidase –[Genbank:WP_004150699.1]) and *Mannheimia haemolytica* (unknown function – [Genbank:WP_021280462.1]).

**S3. Wide variation in the G+C content was identified from the homology groups**

We looked at the BLASTp hits of all homology groups against nr and calculated the %G+C content of all of the genes in the pan-genome to look for genes that had not previously been recorded in *H. parasuis,* or that may have originated from other species*.* We found that 28% of the genes had a %G+C content outside the average of 39.4% G+C content (± 5.4%, which is the standard deviation) for *H. parasuis,* with a minimum of 17.8% and maximum of 67.1% G+C content*.* The variation in the %G+C content of the homology groups based on the syntenic pan-genome order can be seen in Figure S6, alongside a histogram of the G+C content of all genes. Figure S6 showed a wide range of %G+C content within the predicted genes, which may be skewing the average %G+C content. The best matches for the genes outside of the average %G+C range came from a variety of phage and bacterial genera, with *Actinobacillus*, *Mannheimia, Pasteurella* and *Gallibacterium* species containing many of the genes that had not previously been identified in *H. parasuis*. This suggests that horizontal transfer from other species occurs regularly in *H. parasuis*, but as these searches are dependent on the content of NCBI at the time we cannot interpret these data further.

**S4. Second level BAPS analysis**

The second level BAPS analyses identified 18 populations and as before, we compared these second level BAPS populations to the clinical metadata (Figure S7). From this we can see separation based on geography (particularly Denmark and the UK) as well as serovar (particularly serovars 4, 5, 13 and 14). The geographical separation of second level BAPS populations may represent parent populations of *H. parasuis* that have remained in these countries, while some strains have been exported with pigs to other areas to establish new herds. While this second level BAPS analysis shows a better relationship with the clinical metadata it does not fully explain the separation of the population structure.

**S5. High correlation found between capsule loci and serotyping result**

For *H. parasuis,* there are multiple strains being used to represent individual serovars in reference strain sets around the world. We sequenced the strains that differ in name, as well as some that have the same strain name and are used by multiple labs, to see how similar these strains are. Copies of strains 174, C5, H465, IA-84-17975, Nagasaki and SW114, SW140 were identical based on their core and accessory composition from our assemblies and analysis, as can be seen based on their location in the tree of Figure 2. However, the D74 strains representing serovar 9 show variation in both the core and accessory between the strains used in Denmark and Australia. The different strains representing serovars 1 and 7 are not closely related based on their positions in both the core and accessory genome trees but are from the same BAPS population. However, for serovar 10 the strains appear to be distantly related, with one reference strain is found in BAPS population 2 and the other in BAPS population 5.

We investigated the correlation between the capsule loci of the 15 reference strains and other serotyped isolates in our collection (n=117). The nucleotide sequences of the capsule loci from the fifteen serovars were used to perform BLASTn searches against the isolate collection using a cut-off of 90% of the length of the loci and greater than 80% identity. Any strains that had differing results (between serovar and capsule locus) or less than 85% identity between the capsule loci sequences were checked using the Artemis Comparison Tool (ACT) [4]. If the capsule loci were found to match between isolates of the same serovar, the capsule loci (between *funA* and *iscR*) were aligned and the average identity calculated (using alistat [5]). Based on the previous analysis by Howell et al. 2013, we were unable to separate serovars 5 and 12 and so they were grouped together in this analysis. This allowed us to establish that the majority of the serovars had a high association between the capsule locus and the serovar, with 85% identity of the capsule locus for serovar 6 (between *funA* and *iscR*), and greater than 98% identity for serovars 1, 2, 3, 4, 5 or 12, 7, 8, 9, 13 and 15 (Table S5). Importantly, for the different reference strains that are being used to represent each serovar, the capsule loci were found to be identical. This is also supports the hypothesis that the capsule loci are the main determinant of serovar, as the distantly related isolates contain the same capsule locus. Where the sequence of the capsule locus did not match the primary serotyping result, it matched the cross-reaction if one was recorded.

We also found the presence of the capsule loci in all NT isolates (15 of the 117 serotyped isolates) from these BLASTn searches, and have detailed which capsule locus they possess in Table S5. No new capsule loci were identified based on these NT isolates, but indels were found in some strains that may have impacted on the serotyping result. Alternatively the differences between the serotyping results and the capsule loci may be due to differences in production of the capsule during growth or due to an error in the serotyping method itself. We have also predicted the serovar of the remainder of the isolate collection based on the presence of the capsule loci and have found a high number of serovars 4, 5 or 12, 7 and 13 as is seen in the serotyped isolate collection (Figure 1). This can be seen in Figure S3 and then in Figure S8, where the capsule synteny based diagram has been coloured based on the predicted serovar of the isolates. Only two strains from the remainder of the collection did not match to the capsule loci of the 15 reference strains.

**S6. Identification of potential serovar-specific markers from the pan-genome**

While the capsule locus appears to be serovar specific, we were also interested in identifying potential serovar-specific markers outside of this locus. So as a proof of concept, we also used DAPC and glm on the candidates for separating serovars, using iterations of the PCA including between 60-90% of the eigen-values. This allowed us to identify genotypes (either SNPs or genes) that correlated with serovars 4, 5, 7, 12, 13 within our serotyped collection, some of which are within the capsule loci. Our isolate collection had fewer than 5 isolates of serovars 1, 2, 3, 6, 8, 9, 10, 11, 14, and 15 and so we were not able to identify statistically significant markers for these serovars. However, these serovars were useful in the analyses for separating the remaining serovars from the population. For the core genome, DAPC of the presence and absence of genes showed two main groups of serovars clustered together, but individual serovar clusters cannot be seen (Figure S9a). Glms revealed seven significant SNPs for serovars 4 and 5 that differentiate them from the other serovars. The analysis of the accessory genome based on SNPs in association with serovar showed little separation of the serovars in the discriminant function plot shown in Figure S9b (retaining 80% of eigen-values in the PCA). In fact, twenty-three potential serovar-specific genes were identified from the DAPC and glm analysis, five of these candidates were previously identified capsule genes, three phage genes, a transposase, a filamentous haemagglutinin and 13 have unknown functions. This analysis was based on a relatively small subset of strains when considering the number of isolates of each individual serovar, and so with a greater number of the less prevalent serovars, these methods have a higher likelihood of finding further serovar-specific gene markers. They also support the use of the capsule loci as containing serovar specific markers. The results of the DAPC and glm provide potential serovar-specific genes that could be used to design a molecular serotyping assay. The advantages of a molecular test over the original serotyping assays include reduced turnaround time of result and reduced cost, as well as potentially increasing sensitivity and specificity of the assay.

**S7. Limited evidence of reductive evolution found in *H. parasuis***

The process of reductive evolution has been found in virulent isolates of *Klebsiella pneumoniae* [8] and *Shigella* [9]. This process involves the loss of genes or increased numbers of pseudogenes may indicate reductive evolution in a pathogen as it adapts to a new niche or loses anti-virulence genes that interfere with its potential virulence [10, 11]. Therefore we studied the genome size of strains in comparison with their clinical phenotype and serovar as an indication of their virulence, as well as by BAPS populations [12, 13]. There was no evidence of a relationship between genome size and metadata based on our isolate collection, with an average of 2,231 genes predicted for each isolate (Figure S10a-c). In addition there was no difference in the number of pseudogenes between the categories (Figure S10d-f) (18-62 pseudogenes/isolate). As *H. parasuis* could be classed as an opportunistic pathogen it may be at the beginning of the process of reductive evolution and so the accumulation of pseudogenes and inactivation of superfluous genes is not evident yet. On the other hand, this bacterium still occupies multiple environments within the pig and we found isolates that were closely related to clinical isolates in the upper respiratory tract. Therefore it may be possible that a virulent isolate can persist in the upper respiratory tract without causing systemic disease, instead of progressing rapidly to a systemic disease state. The health status of the host also plays an important role in the progression of disease. However, a bacterium that can survive throughout different stages of infection, for example in the upper respiratory tract as well as potentially in the lung, the blood or in other systemic sites around the body, may require a greater repertoire of genes than one that stays within one body site. Based on our data we cannot say that there is a difference in number of genes, but there may be differences in regulation and expression of genes between isolates.

**S8. Genome assembly**

For each isolate, the paired-end reads were mapped to the complete reference genome SH0165 using Stampy (with default settings) [14]. BAM files were created using samtools [15] and compared to the SH0165 complete genome sequence using the Integrative Genome Viewer (IGV) [16]. Each isolate was manually checked for the presence of SNPs, deletions and coverage from the sequencing reads, as a final check to ensure the quality and purity of the isolates before beginning any analyses. Any sequence files failing this control were not included in the analyses. The software Cutadapt was used to remove the adapter sequences that were previously introduced during the library preparation from the sequence reads [17]. Undetermined nucleotides (Ns) were removed from reads and the program Sickle was used to trim the low-quality sequence found at the ends of sequence reads, using the program’s default quality thresholds for the reads [18]. Reads shorter than 31bp after trimming were discarded. Finally, we used Velvet and VelvetOptimiser 2.2.0 [19, 20] to assemble the reads into contiguous sequences (contigs). Assembly parameters were optimised using VelvetOptimiser, which ran through all possible k-mer values from 19 to 71 in increments of 2 [19]. The assemblies were compared to the SH0165 complete genome [21] using BLAST and ACT [4] to look for signs of mis-assembly.

**S9. Homology Group Checks**

Custom python scripts were used to produce Fasta files, alignments and phylogenetic trees of the individual homology groups using muscle and RAxML [22, 23]. These were checked using custom python and R scripts, to determine the F-statistic (using the R package seqinr [24]) for the groups containing multiple genes from the same isolate, and these were used in combination with the phylogenetic trees to determine if a homology group should be separated into multiple groups. For example a homology group was split up if a high F-statistic was found with a low identity, with the corresponding phylogenetic tree showing duplicates in separate groups on the tree. Where duplicates were due to proteins split into multiple coding sequences, these were removed to separate alignments. The remainder of the groups were assessed using alistat [5] to look at the alignment lengths and minimum identity between pair wise alignments within the homology groups to identify both pseudogenes and homology groups that required further separation. Pseudogenes were defined as within 80% of the modal alignment length of the group, and were calculated and split using custom python scripts [25, 26]. No further splitting of the sequences based on length was performed within these pseudogene groups. Prokka annotation of the draft genome sequences was also used to compare the predicted functions within the homology groups to look for further anomalous groupings [27]. Finally a BLAST database of all of the homology groups was created and the longest sequence from each homology group was selected to query the database to search for any false negatives within the homology groups, to see if any groups had been unnecessarily split up and should be combined. After this series of alterations to the homology groups a final python script was used to ensure that all proteins were still present within the homology groups.

**Supplemental References:**

1. Tatusov RL, Natale D a, Garkavtsev I V, Tatusova T a, Shankavaram UT, Rao BS, Kiryutin B, Galperin MY, Fedorova ND, Koonin E V: **The COG database: new developments in phylogenetic classification of proteins from complete genomes.** *Nucleic Acids Res* 2001, **29**:22–8.

2. Tatusov RL, Galperin MY, Natale D a, Koonin E V: **The COG database: a tool for genome-scale analysis of protein functions and evolution.** *Nucleic Acids Res* 2000, **28**:33–6.

3. Howell KJ, Weinert L a, Luan S-L, Peters SE, Chaudhuri RR, Harris D, Angen O, Aragon V, Parkhill J, Langford PR, Rycroft AN, Wren BW, Tucker AW, Maskell DJ: **Gene content and diversity of the loci encoding biosynthesis of capsular polysaccharides of the fifteen serovar reference strains of *Haemophilus parasuis*.** *J Bacteriol* 2013, **195**:4264–73.

4. Carver T, Berriman M, Tivey A, Patel C, Böhme U, Barrell BG, Parkhill J, Rajandream M-A: **Artemis and ACT: viewing, annotating and comparing sequences stored in a relational database.** *Bioinformatics* 2008, **24**:2672–6.

5. Eddy SR: **SQUID - C function library for sequence analysis**. 2005.

6. Roberts IS: **The biochemistry and genetics of capsular polysaccharide production in bacteria.** *Annu Rev Microbiol* 1996, **50**:285–315.

7. Whitfield C: **Biosynthesis and assembly of capsular polysaccharides in *Escherichia coli*.** *Annu Rev Biochem* 2006, **75**:39–68.

8. Brisse S, Fevre C, Passet V, Issenhuth-Jeanjean S, Tournebize R, Diancourt L, Grimont P: **Virulent clones of *Klebsiella pneumoniae*: identification and evolutionary scenario based on genomic and phenotypic characterization.** *PLoS One* 2009, **4**:e4982.

9. Peng J, Yang J, Jin Q: **The molecular evolutionary history of *Shigella spp*. and enteroinvasive *Escherichia coli*.** *Infect Genet Evol* 2009, **9**:147–52.

10. Bliven K a, Maurelli AT: **Antivirulence genes: insights into pathogen evolution through gene loss.** *Infect Immun* 2012, **80**:4061–70.

11. Merhej V, Georgiades K, Raoult D: **Postgenomic analysis of bacterial pathogens repertoire reveals genome reduction rather than virulence factors.** *Brief Funct Genomics* 2013, **12**:291–304.

12. Ochman H: **Genes Lost and Genes Found: Evolution of Bacterial Pathogenesis and Symbiosis**. *Science (80- )* 2001, **292**:1096–1099.

13. Maurelli a T, Fernández RE, Bloch C a, Rode CK, Fasano a: **“Black holes” and bacterial pathogenicity: a large genomic deletion that enhances the virulence of Shigella spp. and enteroinvasive *Escherichia coli*.** *Proc Natl Acad Sci U S A* 1998, **95**:3943–8.

14. Lunter G, Goodson M: **Stampy : A statistical algorithm for sensitive and fast mapping of Illumina sequence reads**. 2011:936–939.

15. Li H, Handsaker B, Wysoker A, Fennell T, Ruan J, Homer N, Marth G, Abecasis G, Durbin R: **The Sequence Alignment/Map format and SAMtools.** *Bioinformatics* 2009, **25**:2078–9.

16. Robinson JT, Thorvaldsdottir H, Winckler W, Guttman M, Lander ES, Getz G, Messirov JP: **Integrative Genomics Viewer**. *Nat Biotechnol* 2011, **29**:24–26.

17. Martin M: **Cutadapt removes adapter sequences from high-throughput sequencing reads**. *EMBnet.journal* 2011, **17**:10–12.

18. Joshi N, JN F: **Sickle: A sliding-window, adaptive, quality-based trimming tool for FastQ files (Version 1.29)**. 2011.

19. Zerbino DR, Birney E: **Velvet: algorithms for de novo short read assembly using de Bruijn graphs.** *Genome Res* 2008, **18**:821–9.

20. Gladman S: **VelvetOptimiser**. 2009.

21. Xu Z, Yue M, Zhou R, Jin Q, Fan Y, Bei W, Chen H: **Genomic characterization of *Haemophilus parasuis* SH0165, a highly virulent strain of serovar 5 prevalent in China.** *PLoS One* 2011, **6**:e19631.

22. Edgar RC: **MUSCLE: multiple sequence alignment with high accuracy and high throughput.** *Nucleic Acids Res* 2004, **32**:1792–7.

23. Stamatakis A: **RAxML-VI-HPC: maximum likelihood-based phylogenetic analyses with thousands of taxa and mixed models.** *Bioinformatics* 2006, **22**:2688–90.

24. Penel MS: **Package “ seqinr .”**2012.

25. Ochman H, Davalos LM: **The nature and dynamics of bacterial genomes.** *Science* 2006, **311**:1730–3.

26. Lerat E, Ochman H: **Recognizing the pseudogenes in bacterial genomes.** *Nucleic Acids Res* 2005, **33**:3125–32.

27. Seemann T: **Prokka: rapid prokaryotic genome annotation.** *Bioinformatics* 2014:1–2.
