# Supplementary figures and images for: The use of genome wide association methods to investigate pathogenicity, population structure and serovar in Haemophilus parasuis
Source: BMC Genomics. 2014 Dec 24;15:1179. doi: 10.1186/1471-2164-15-1179 (PMC4532294; doi:10.1186/1471-2164-15-1179)

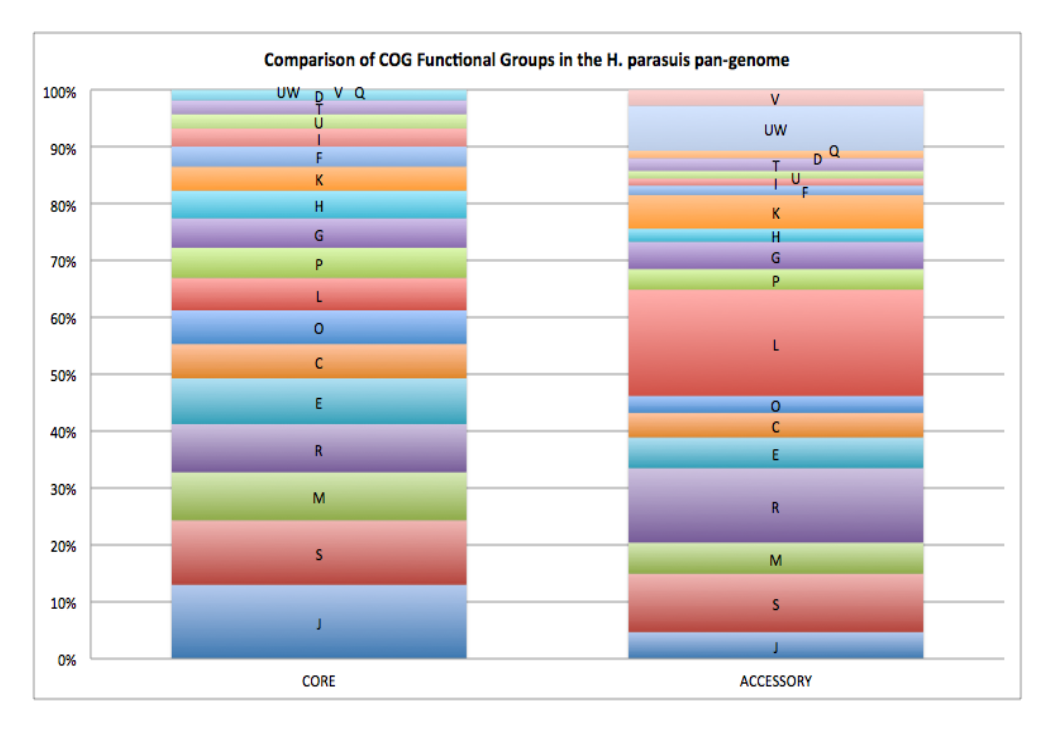

Supplement: Supplementary file 3 — Additional file 3: Figure S1: Synteny of the pan-genome created based on the SH0165 complete genome, with black areas showing presence of a gene, and white areas absence. Areas of variation in the synteny with predicted phage genes are highlighted in red, areas with high levels of genes of unknown function in green. Numbers indicate regions of variation. Antibiotic resistance gene locations are represented by orange lines. (PNG 143 KB) [file 12864_2014_7083_MOESM3_ESM.png]

1

2

3

4

5

6

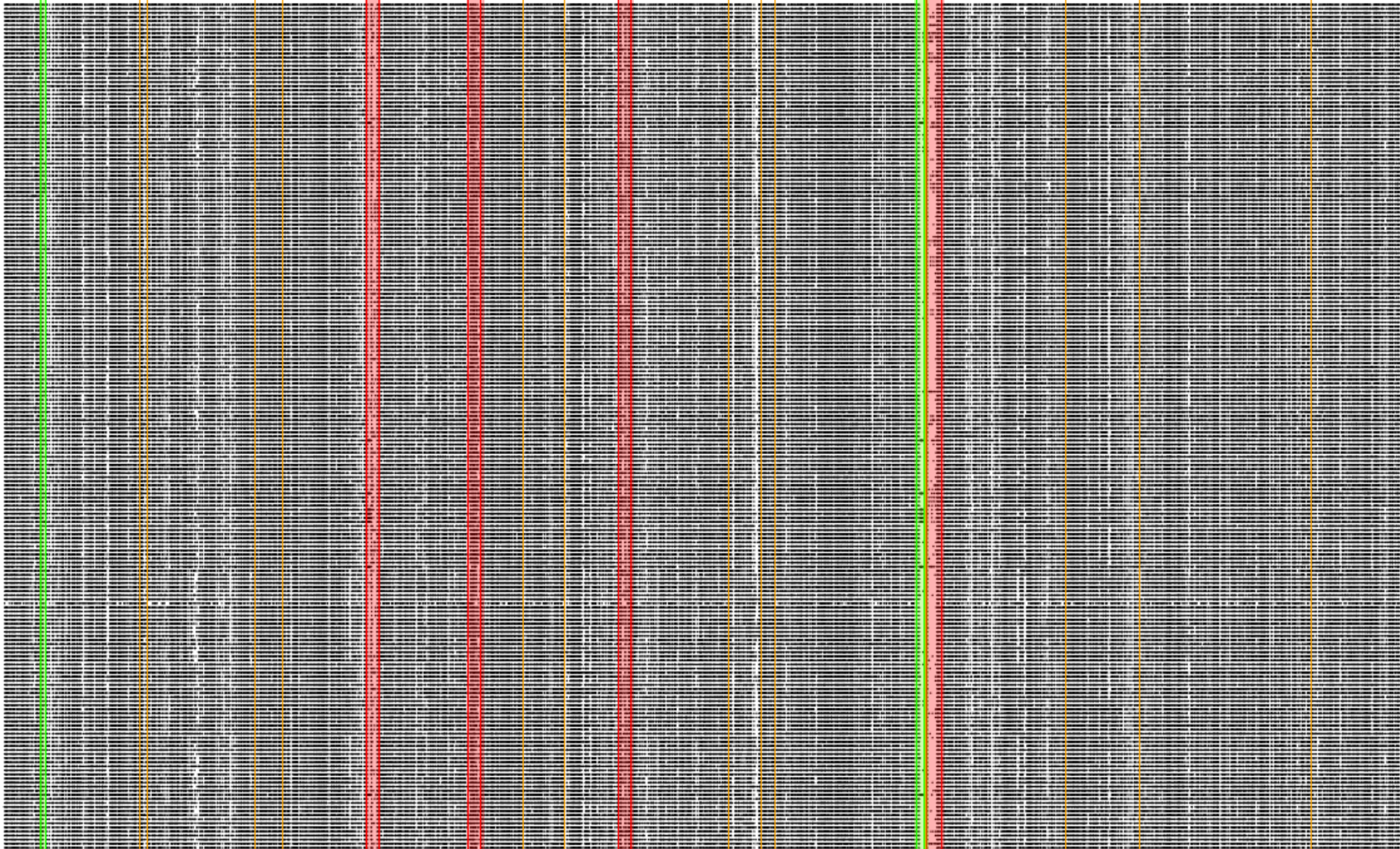

Supplement: Supplementary file 4 — Additional file 4: Figure S2: Synteny of the capsule loci of Haemophilus parasuis. Rows represent the isolates from the collection and black diamonds represent the presence of genes. Each isolate was found to possess a capsule locus, isolates have been ordered by their similarity in the pattern. The beginning and end of the locus are conserved but a lot of variation can be seen within the locus. (PDF 733 KB) [file 12864_2014_7083_MOESM4_ESM.pdf]

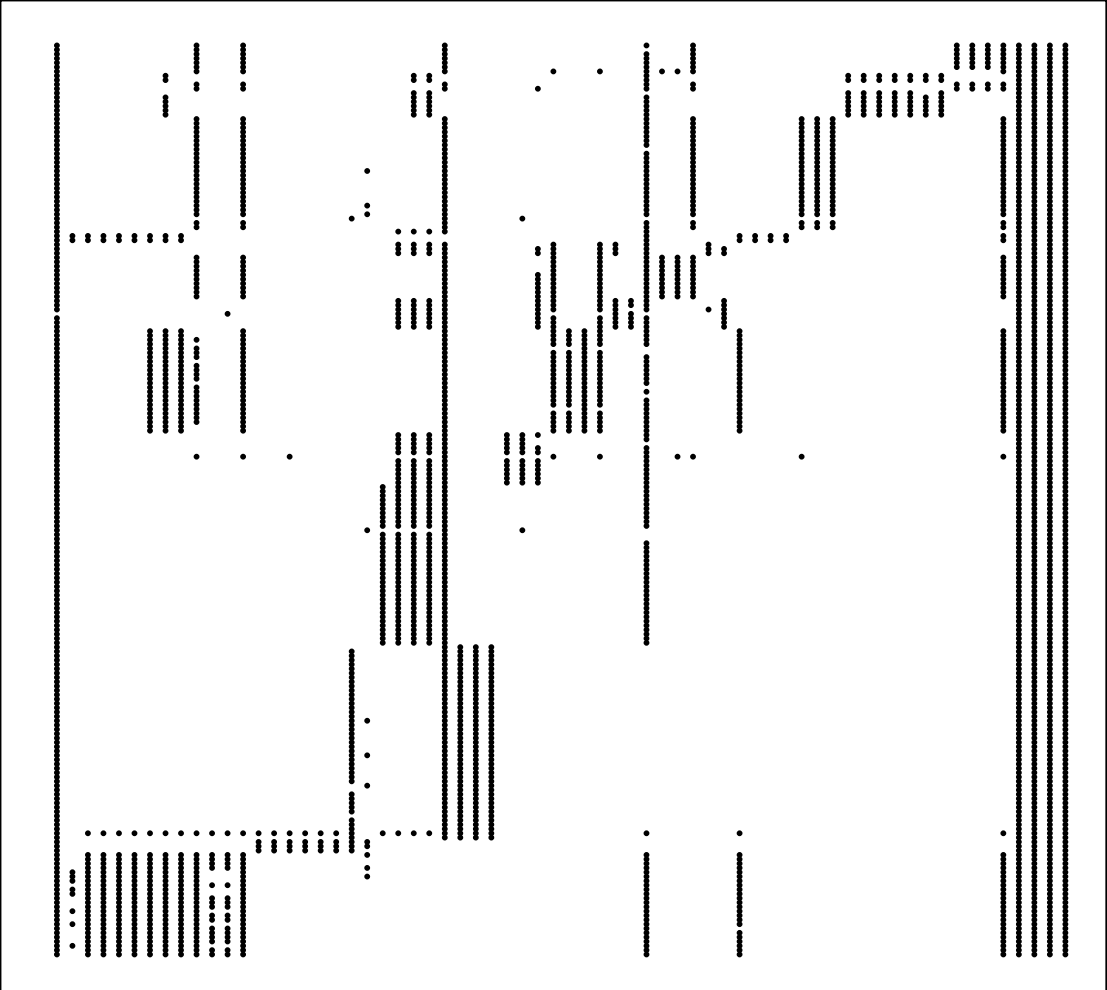

Supplement: Supplementary file 5 — Additional file 5: Figure S3: Bayesian Analysis of Population Structure of 212 isolates of H. parasuis. Each row in the figure is a strain and each column is a SNP with sites ordered to maximize visual separation of the five distinct clusters. Horizontal black lines are drawn to separate the clusters obtained in the first level of clustering. (PDF 14 KB) [file 12864_2014_7083_MOESM5_ESM.pdf]

**G+C content of syntenic pan-genome**

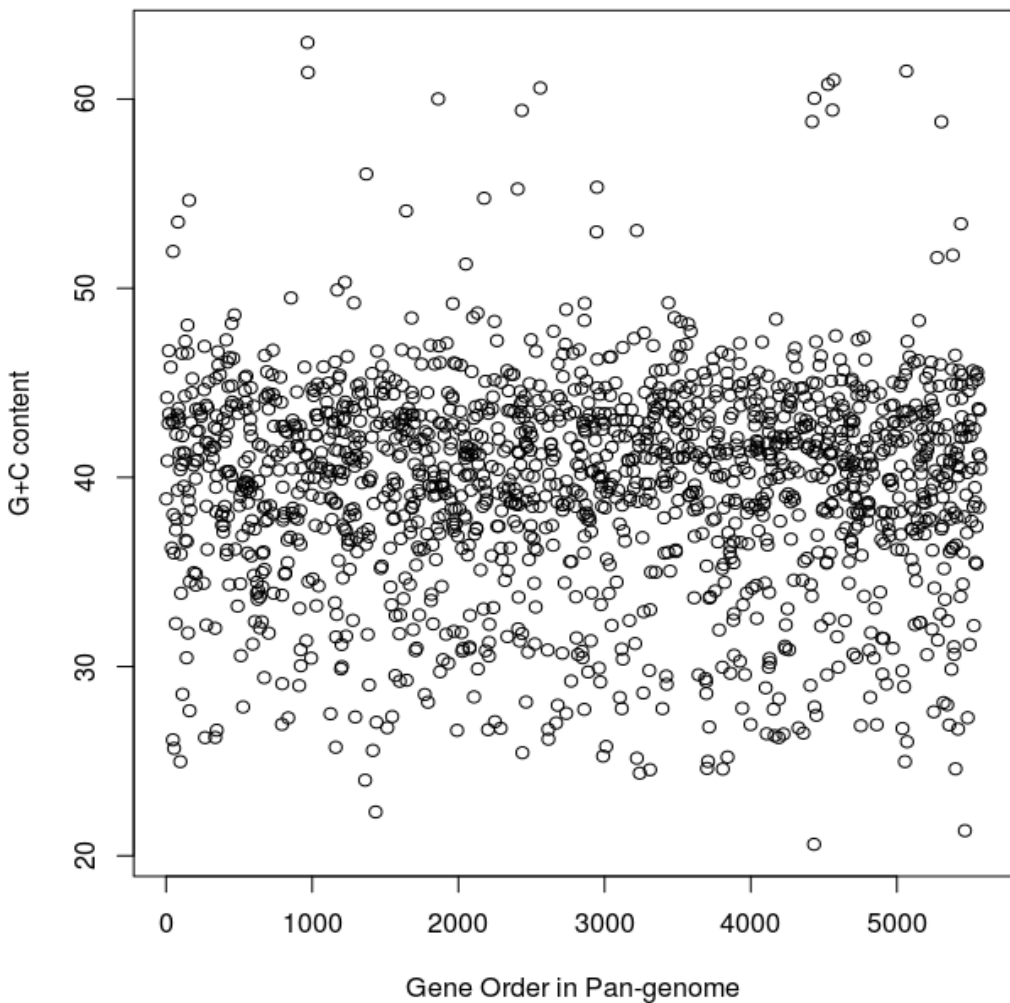

**G+C content of all genes**

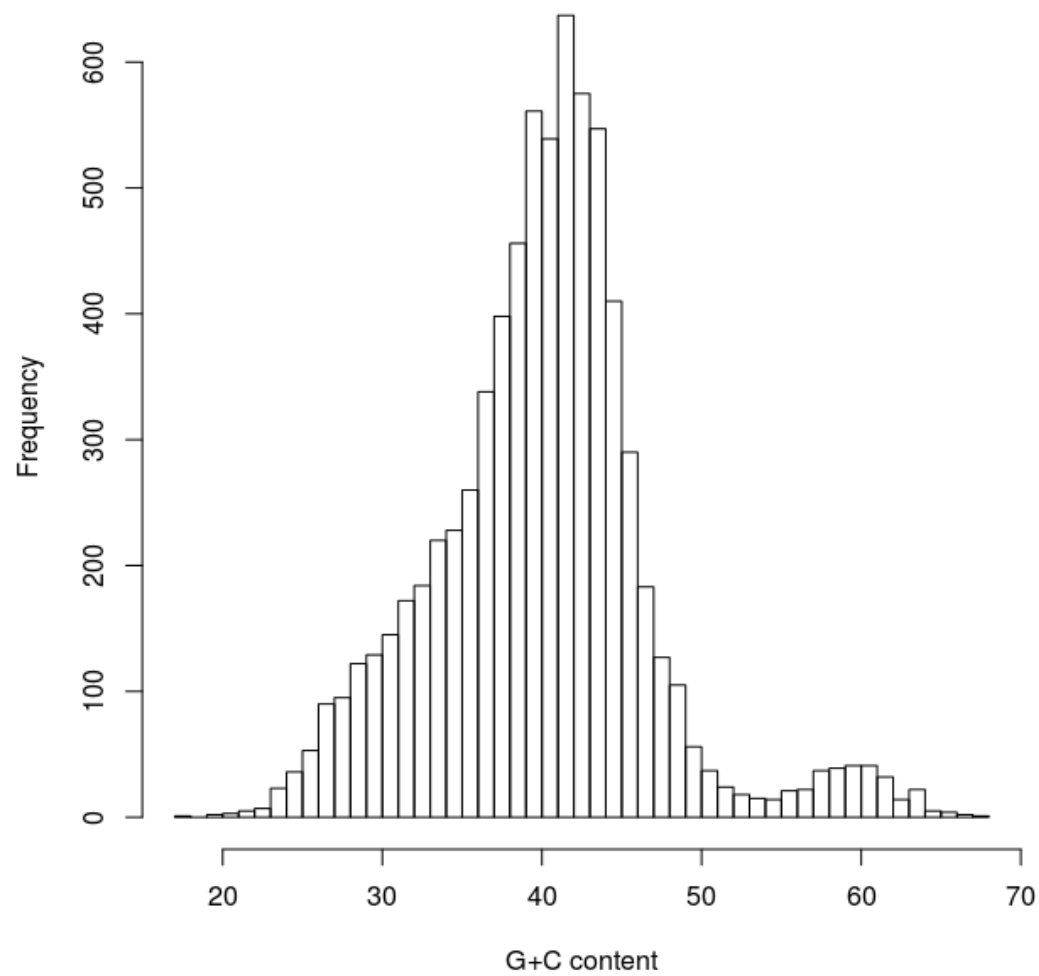

Supplement: Supplementary file 6 — Additional file 6: Figure S4: Discriminant analysis of principal components applied to the BAPS populations and two clades of H. parasuis (80% eigenvalues retained for the PCA), all eigenvalues were retained for the discriminant analysis. Plots a and c show the first two axes of the discriminant function while b and d show the first axis only. Separation along the axes suggests that genetic differences are present between the BAPS populations and clades. A large degree of separation can be seen from the both accessory genome and the core genome, and is far more pronounced for the two clades of H. parasuis. (PDF 169 KB) [file 12864_2014_7083_MOESM6_ESM.pdf]

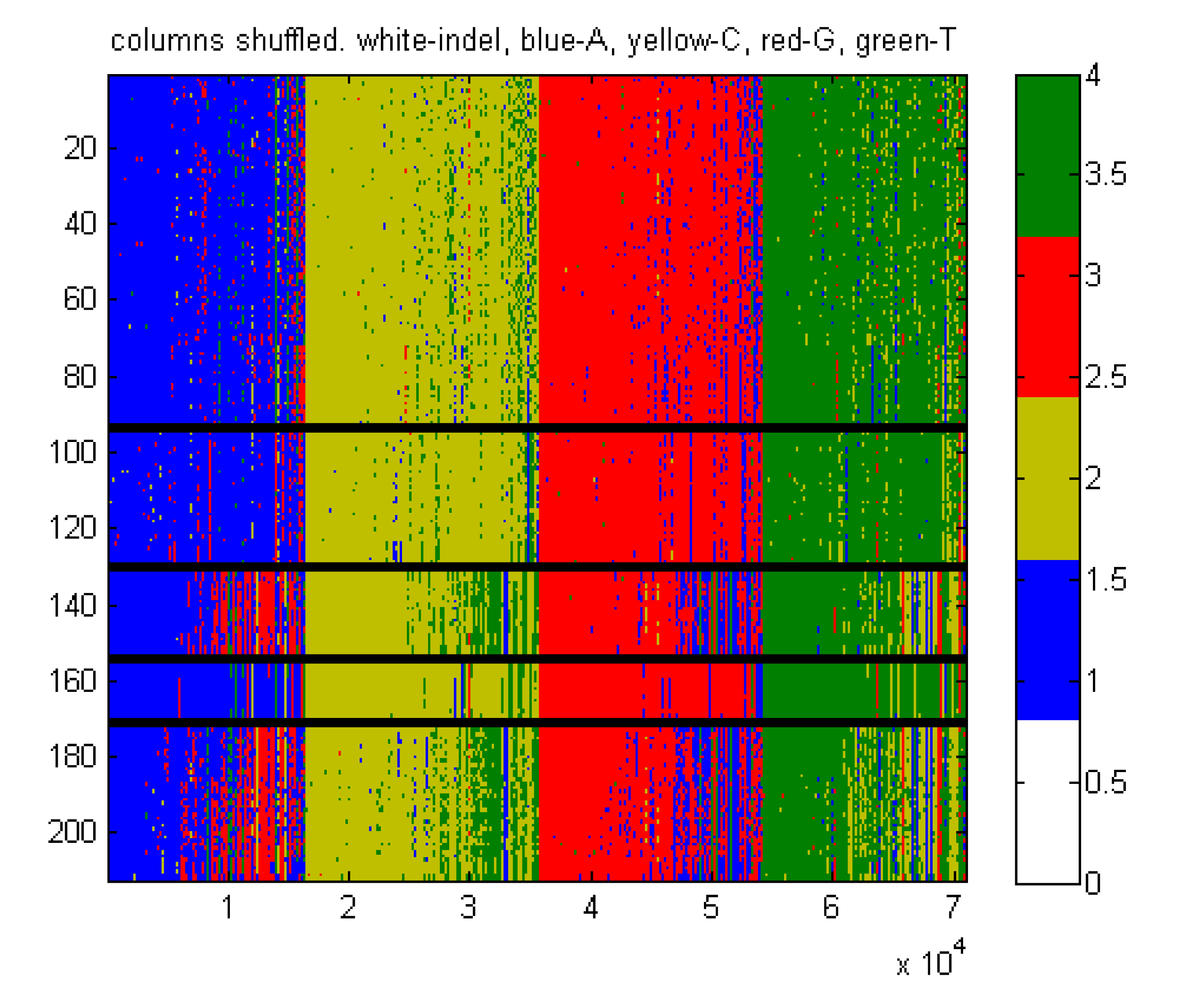

Supplement: Supplementary file 7 — Additional file 7: Figure S5: Comparison of the COG functional groups found in the core and accessory genome of H. parasuis found at greater than 1%. Several differences in proportions of COG groups can be seen between the core and the accessory genome. The core genome shows a greater proportion of proteins involved in translation, coenzyme metabolism as well as post translational modification over the accessory genome. A greater proportion of cell wall/membrane biogenesis transcription, replication and repair and cell motility, defence mechanisms as well as proteins of unknown function were identified in the accessory genome. CELLULAR PROCESSES AND SIGNALING -[D] Cell cycle control, cell division, chromosome partitioning, [M] Cell wall/membrane/envelope biogenesis, [O] Post-translational modification, protein turnover, and chaperones, [T] Signal transduction mechanisms, [U] Intracellular trafficking, secretion, and vesicular transport, [V] Defense mechanisms, [W] Extracellular structures. INFORMATION STORAGE AND PROCESSING - [J] Translation, ribosomal structure and biogenesis, [K] Transcription, [L] Replication, recombination and repair. METABOLISM- [C] Energy production and conversion, [E] Amino acid transport and metabolism, [F] Nucleotide transport and metabolism, [G] Carbohydrate transport and metabolism, [H] Coenzyme transport and metabolism, [I] Lipid transport and metabolism, [P] Inorganic ion transport and metabolism, [Q] Secondary metabolites biosynthesis, transport, and catabolism. POORLY CHARACTERIZED - [R] General function prediction only, [S] Function unknown. If a gene cannot be classified by a singular COG category then multiple categories can be used, e.g. UW represents proteins involved in intracellular trafficking [U] and extracellular structures [W]. (PNG 115 KB) [file 12864_2014_7083_MOESM7_ESM.png]

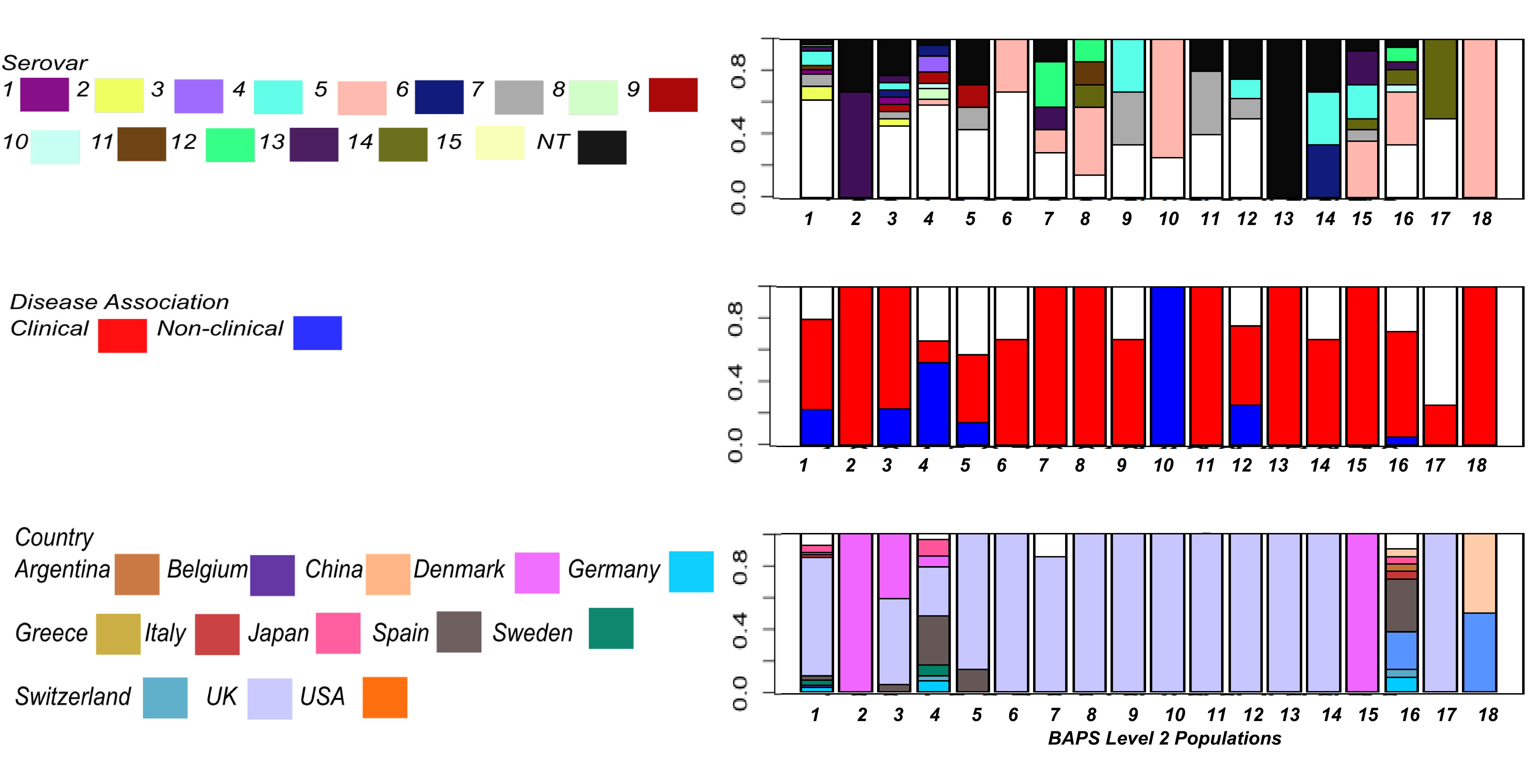

Supplement: Supplementary file 8 — Additional file 8: Figure S6: G+C content of the H. parasuis pan-genome. Plot a) shows the variation of the G+C content based on the syntenic order of the pan-genome, while plot b) shows a histogram of the variation in all predicted genes. (PNG 317 KB) [file 12864_2014_7083_MOESM8_ESM.png]

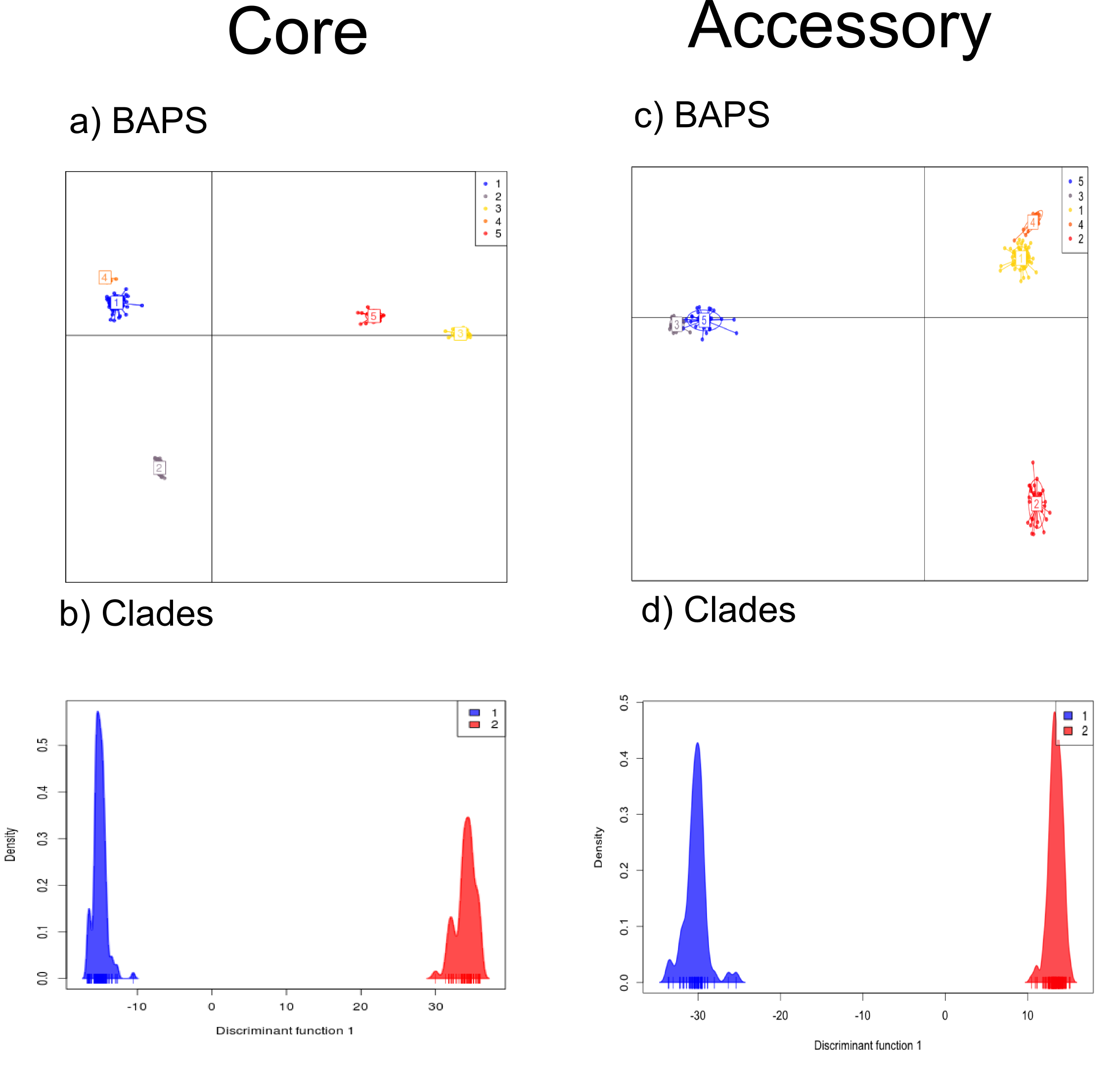

Supplement: Supplementary file 9 — Additional file 9: Figure S7.: BAPS populations level 2 comparison to clinical metadata including serovar, disease association and country. Greater separation of serovars (particularly for serovar 5), disease association and geography (for the UK and Denmark) can be seen based on these more refined populations. (PNG 201 KB) [file 12864_2014_7083_MOESM9_ESM.png]

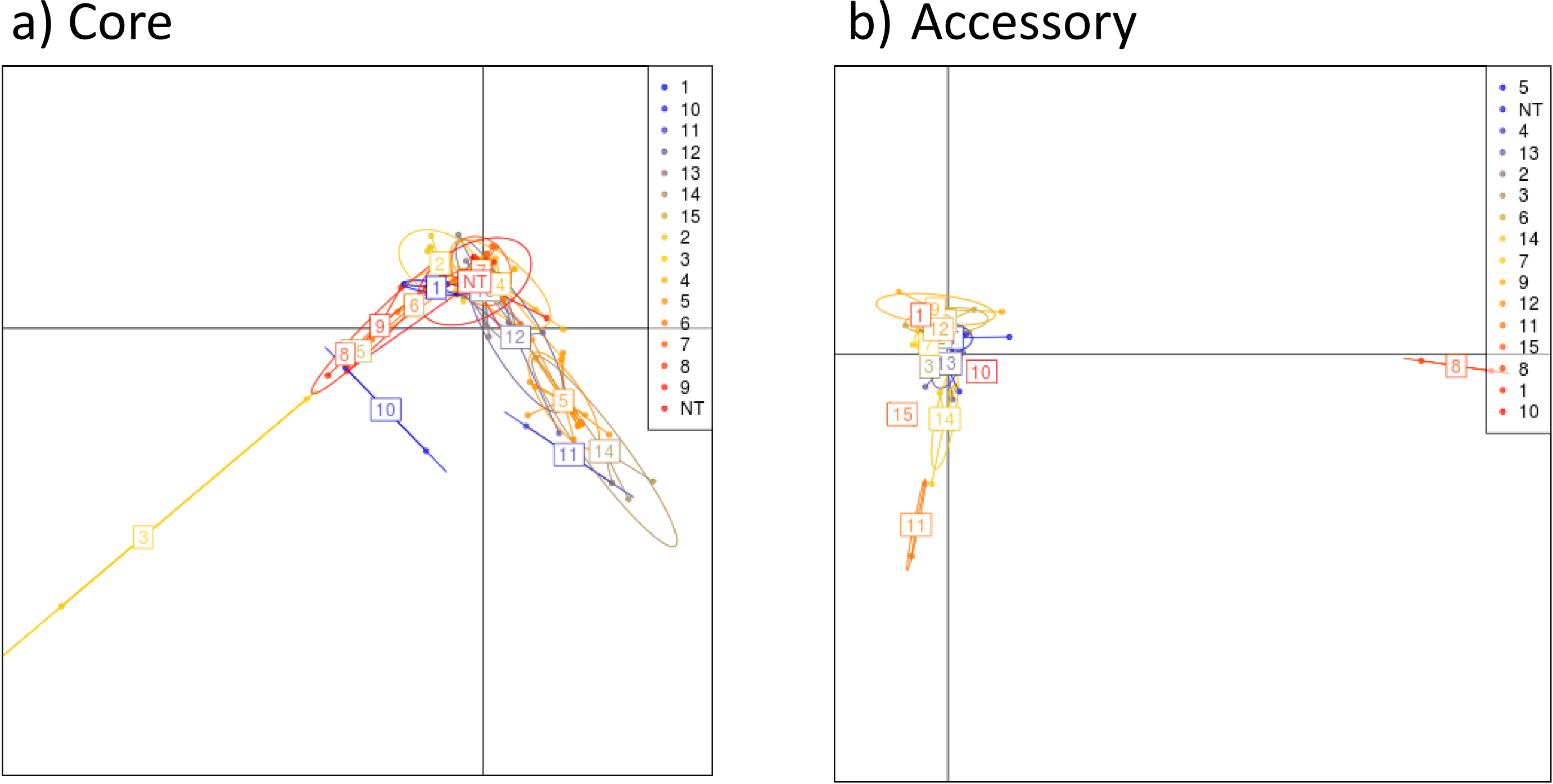

Supplement: Supplementary file 12 — Additional file 12: Figure S8: Synteny of the capsule loci of Haemophilus parasuis. Rows represent the isolates from the collection, coloured diamonds represent the presence of genes. Each isolate was found to possess a capsule locus, isolates have been ordered by their predicted serovar. The beginning and end of the locus are conserved but a lot of variation can be seen within the locus. The pattern based on the predicted serovars does fit with the presence and absence of the genes for the majority of isolates. (PNG 156 KB) [file 12864_2014_7083_MOESM12_ESM.png]

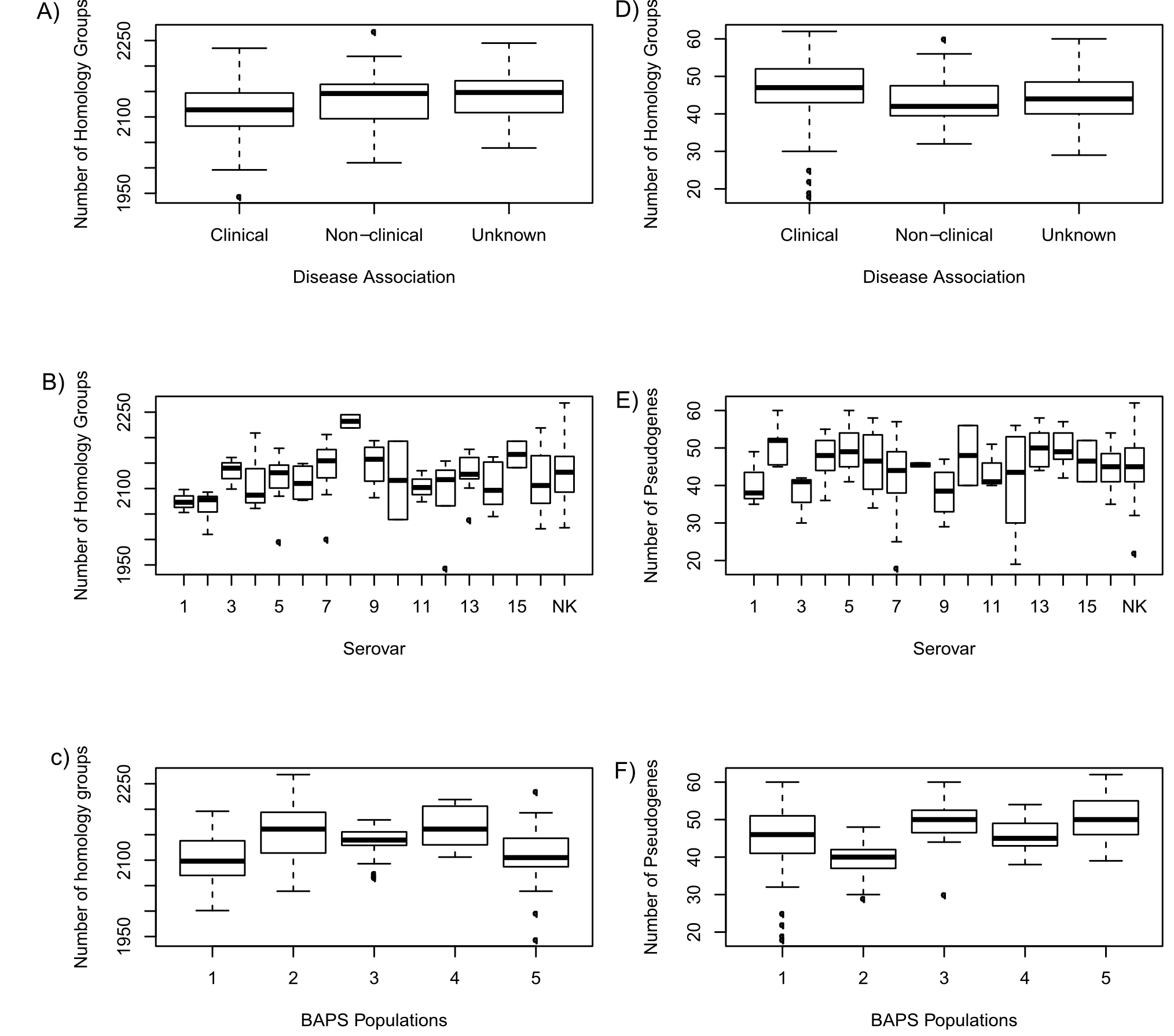

Supplement: Supplementary file 13 — Additional file 13: Figure S9: Discriminant Analysis of Principal Components applied to the core genome and accessory for H. parasuis serotyped strains, retaining 80% of the PCA eigenvalues. A) Separation of the serovars into two main groups can be seen based on the first two axes of the discriminant function. B) Greater separation of the discriminant function of serotyped strains by serovar can be seen from the core in comparison to the accessory. However these serovars have a low number of strains within these groups. (PNG 180 KB) [file 12864_2014_7083_MOESM13_ESM.png]

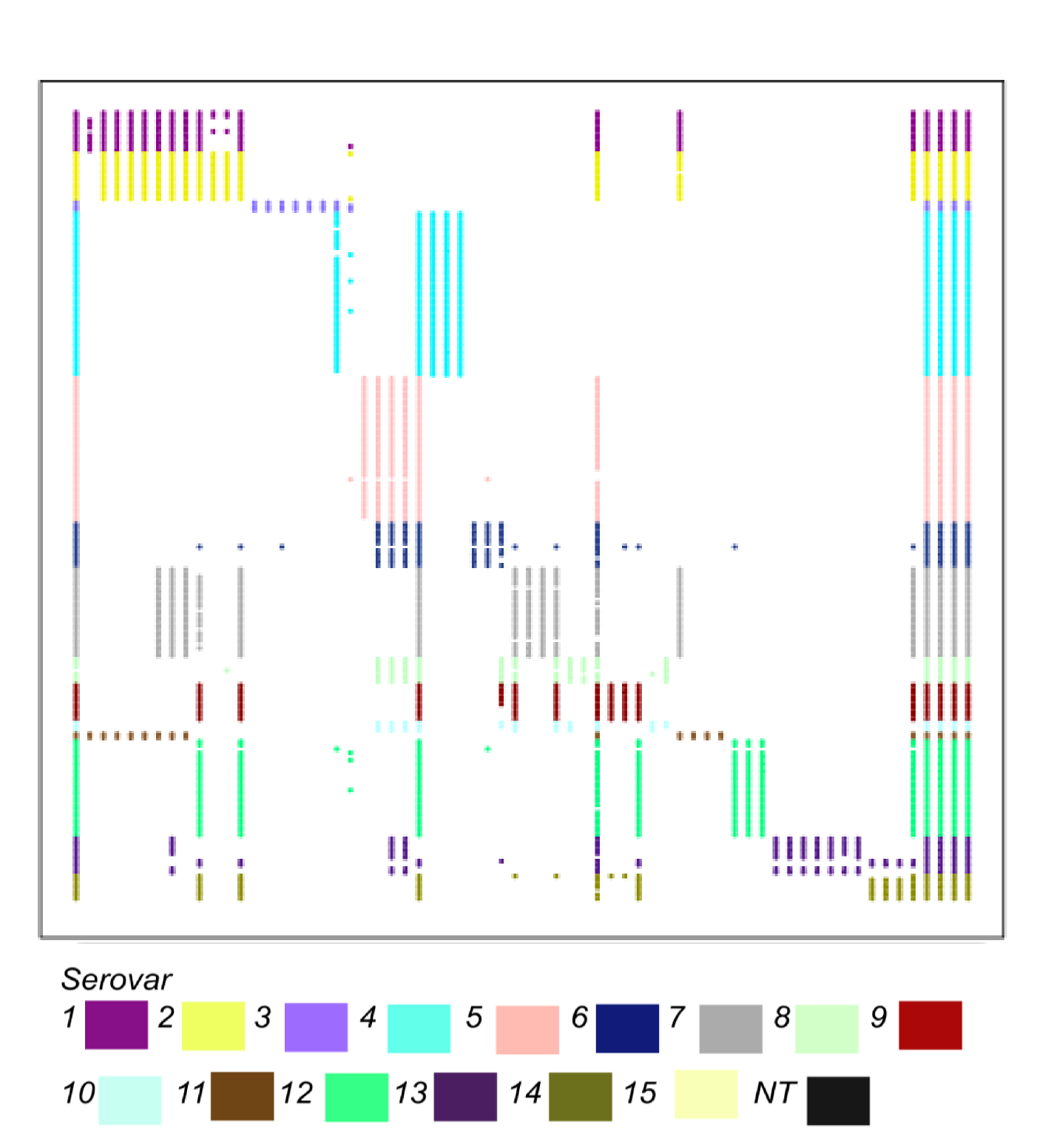

Supplement: Supplementary file 16 — Additional file 16: Figure S10: Comparison of genome size (based on the number of homology groups and pseudogenes) using box and whisker plots. No difference can be seen in either genome size or number of pseudogenes for disease association, serovar or BAPS populations. (PNG 133 KB) [file 12864_2014_7083_MOESM16_ESM.png]
